# Exceptional coprolite association from the Early Cretaceous continental Lagerstätte of Las Hoyas, Cuenca, Spain

**DOI:** 10.1371/journal.pone.0196982

**Published:** 2018-05-23

**Authors:** Sandra Barrios-de Pedro, Francisco José Poyato-Ariza, José Joaquín Moratalla, Ángela D. Buscalioni

**Affiliations:** 1 Unidad de Paleontología, Departamento de Biología, Edificio de Biología, Universidad Autónoma de Madrid, Cantoblanco, Madrid, Spain; 2 Instituto Geológico y Minero de España (Museo Geominero), Madrid, Spain; Indiana University Bloomington, UNITED STATES

## Abstract

Coprolites are some of the most abundant fossils at the Las Hoyas site, a well-known Early Cretaceous Konservat-Lagerstätte located in Cuenca, central Spain. The coprolite association is described, introducing taphonomic features and sedimentological properties. This study is based on a subsample of 433 fossils selected from some 2000 specimens collected. The taphonomic features of the coprolites show that their integrity, absence of desiccation marks, and volume are congruent with faeces produced and deposited in an aquatic ecosystem, which were immediately covered by microbial mats. The highest abundance of coprolites, 96%, occurs in layers linked to the presence of microbial mats. Consequently, it is likely that coprolites are taphonomically autochthonous. A dichotomous key has been made in order to delimit the morphotypes. The key is based on (1) presence/absence of spiral marks, (2) morphology of coprolite ends, including polarity, expansion, and surface, and (3) overall shape, outline, diameter, and constrictions. Twelve different morphotypes are distinguished: spiral, circular, irregular, elongated, rosary, ellipsoidal, cylinder, bump-headed lace, fir-tree, cone, straight lace, and thin lace. The association is dominated by thin-lace and cylinder morphotypes. The sizes, inclusions, and EDX analyses indicate that the Las Hoyas coprolites correspond mostly to carnivorous producers with ichthyophagous diets, as crocodiles, urodelans and different kind of fishes.

## Introduction

Coprolites are fossilized faeces belonging to a group of ichnofossils called bromalites [1]; the term ‘coprolite’ was defined by Buckland in 1829 [2]. Thulborn [3] (p.342) defined 'coprolite' as “a fecal mass that fossilized after having been removed from the body of an animal”. Previous investigations on coprolites have included studies on their external morphology [3] and their contents, such as bones [4,5], insect remains [6], even wood and muscles [7,8]. In general, most contributions have looked for the identification of the faecal mass producer and its diet, mostly among extinct vertebrates (for example, [4,9–13]). Recent studies have also involved destructive techniques such as the use of isotope analysis [10,14,15], gas chromatographic-mass spectrometry, and specific lipid biomarkers [16–18] to infer, for example, the diet of the producer and even its trophic level in the food chain. These studies integrate descriptions that begin with non-destructive protocols followed by destructive techniques on the same specimens, with the aim of combining the morphology of the coprolites with their corresponding contents, the coprolite fabric, and their chemical composition [19]. Eventually, coprolites can provide direct information about the feeding strategy and diet of the animals that produced them and, as a consequence, indirect information about their feeding interactions, predator-prey relationships, faunal abundance and possible trophic chains of ancient ecosystems (e.g., [1,4,9,13,20–25]). In this first stage, we describe the exceptional assemblage of Early Cretaceous (Barremian) coprolites from Las Hoyas Konservat-Lagerstätte in order to understand the variety and disparity of the coprolite association. This will lead to determination of some biological affinities among the coprolites and to indicate how they are integrated into the ecosystem where they were produced [26,27].

Many of the coprolite-rich Mesozoic assemblages correspond to transitional and marine depositional environments from the Triassic [28,29] and the Cretaceous [27,30,31]. There are famous continental deposits with important tetrapod associations from the Triassic [32] and the Cretaceous [33–36] (see [37] for details). However, there are not many fluvial and lacustrine Mesozoic deposits with rich coprolite associations. The Greenland Kap Stewart Formation [38], with about five hundred specimens, and the Csehbánya in Hungary [39], with more than two thousand coprolites accumulated in a small area, are good examples of rich fluvial and lacustrine coprolite localities. In this sense, the Early Cretaceous locality of Las Hoyas provides another exceptional example of a locality with a rich and extremely varied coprolite association (more than two thousand coprolites collected) from a lacustrine carbonate inland wetland ecosystem [40]. A subsample of 433 coprolites was selected in order to describe their morphological disparity and preservation patterns.

The biodiversity count of the Las Hoyas body fossil record comprises 118 families and 201 species. The diversity is composed by a mixture of fully aquatic, amphibian, and terrestrial organisms [40–43]. Animals constitute 77% of the total diversity at specific taxonomic level, and at their higher taxonomic rank they consist of Annelida, Nematoda, Mollusca, Arachnida, Myriapoda, Ostracoda, Malacostraca, Hexapoda, and Vertebrata. Insects are the most diverse group, as they represent 36% of the total number of species recorded in the locality. The dominant fauna corresponds to obligate aquatic organisms (i.e., ostracods, gastropods, bivalves, shrimps, aquatic insects, fishes, perennibranchiate salamanders, and frogs). Fish are, by far, the most abundant vertebrate fossils: they are taxonomically diverse, and their record includes different ontogenetic stages. Other aquatic organisms are salamanders, crocodiles, and turtles. Terrestrial organisms are numerically scarce in the Las Hoyas fossil record, but include a diverse group that comprises arachnids, myriapods, insects, an albanerpetontid amphibian, lizards, non-avian dinosaurs, and a mammal. Our approach attempts to test the hypothesis that the faeces were produced mostly by the aquatic animals that inhabited the ecosystem, and to recognize and relate the different types of feeding strategies.

## Sedimentological and environmental context

**Fig 1:**
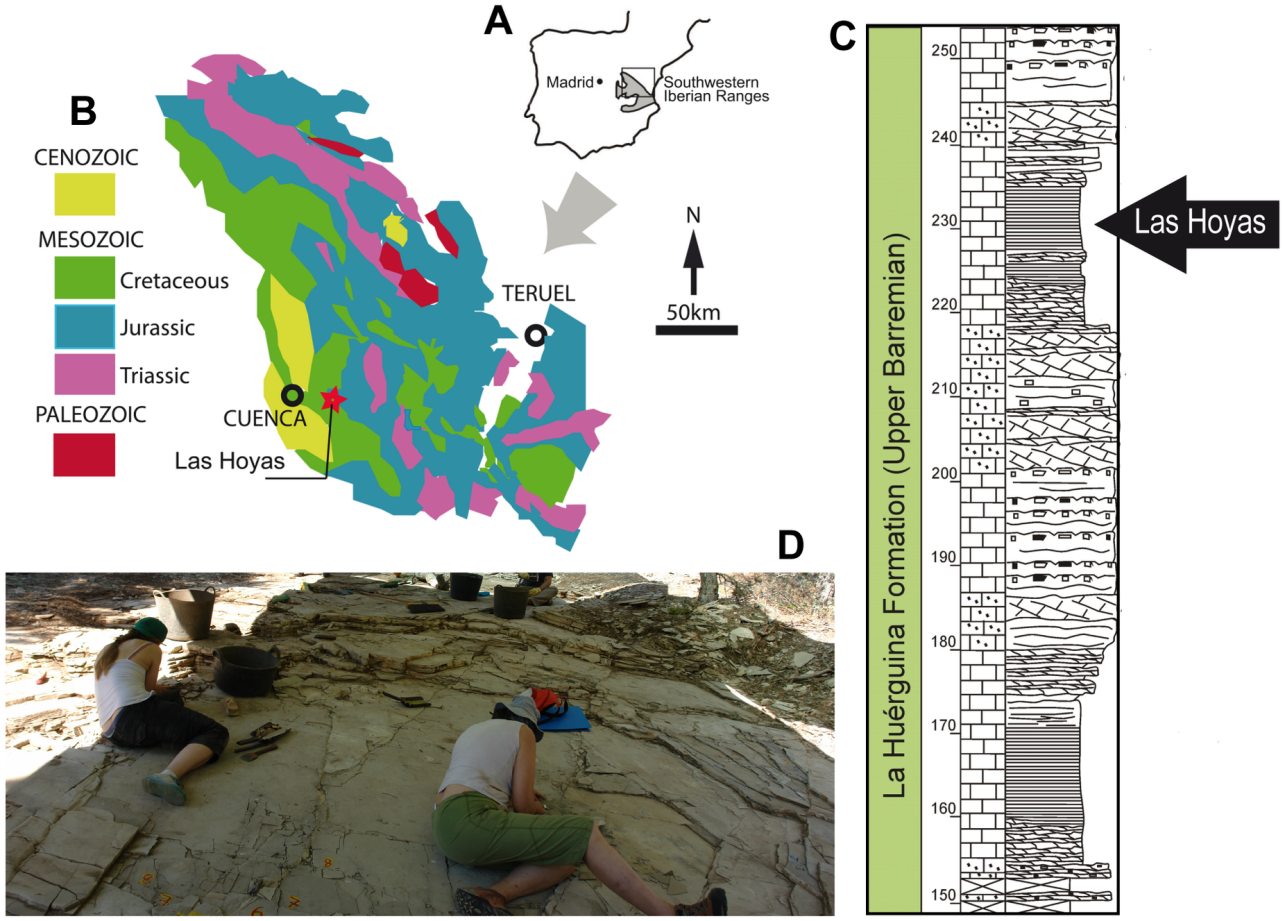
Environmental context of Las Hoyas. **A**. Location of the southwestern sector of the Iberian Ranges. The frame encloses the area represented in **B**, where the locality of Las Hoyas, upper Barremian in age, is placed. **C**. Stratigraphic log of the La Huérguina Formation at the Las Hoyas parastratotype section according to [43]. The arrow points to the finely laminated limestones deposited in Las Hoyas. **D**. Overview of a sampling square at the locality.

The Las Hoyas fossil site is part of the La Huérguina Formation, which records upper Barremian continental sedimentation in the southwestern Iberian Basin (Serranía de Cuenca, Spain) (Fig 1A and 1B). The age of the site has been determined on the basis of the charophyte and ostracod association [44]. The fossiliferous locality consists of finely-laminated limestones (Fig 1C and 1D) composed almost entirely of calcium carbonate [43,45,46]. Las Hoyas has been interpreted as a freshwater carbonatic environment without any marine influence [47,48], regulated by a seasonal subtropical climate in a lacustrine to palustrine wetland subsystem. The wetland was drained by carbonate-rich water, probably fed by groundwater and/or karstic aquifers [49,50]. The general landscape was characterized by a low-relief karstic terrain with a flat topography in which patchy mosaic environments occurred, sheltered by a variety of different vegetation and soils mingled into flooded plains, ponds, small lakes, channels, and sloughs. The watered areas were shallow, their bottoms covered by microbial mats [51,52], and were subjected to seasonal cyclical oscillations in the water level [45,46].

### Microfacies associations in the laminated limestones

**Fig 2:**
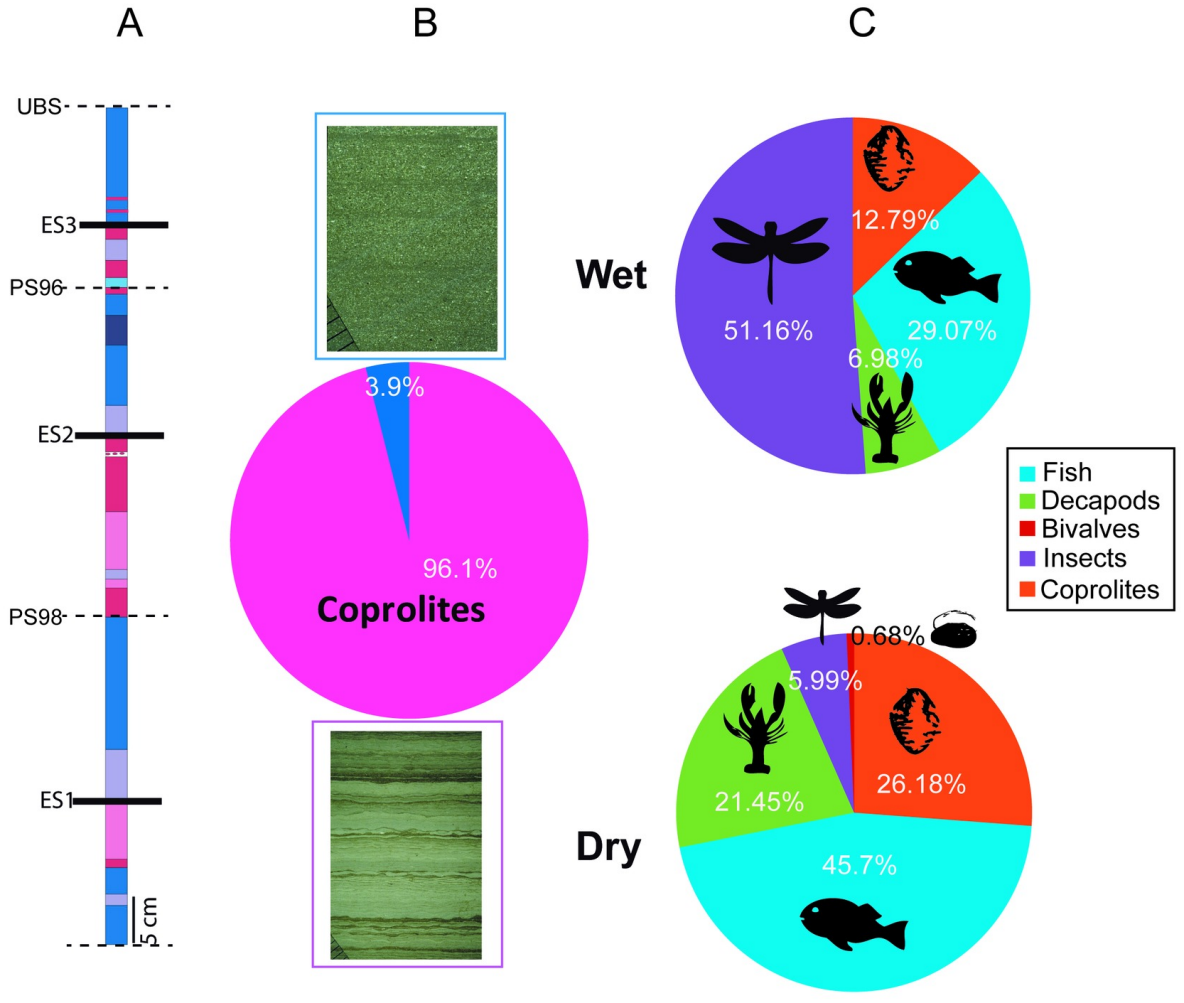
Relative abundance of fossils in wet and dry microfacies. **A.** Stratigraphic log of the sampling squares, showing the sequence of wet and dry microfacies found throughout three Elementary Cycles [40]. The limits (ES) of the PS96, PS98 and UBS sampling squares have been represented. **B.** Coprolite abundance in wet (blue) and dry (pink) facies (N = 282), and one example of each type of microfacies under petrographic microscope. Scale division 1 mm. **C.** Charts with the percentage of relative abundance of bivalves, crustaceans, insects, fishes, and coprolites found in wet (N = 74) and dry (N = 836) facies associations.

The Las Hoyas laminated limestones show two basic microfacies associations [40,46]. These microfacies represent two extremes of a set of transitional microfacies between them. One of these extremes is made up of positively graded millimetric laminae, formed by underflow currents and decantation of allochthonous detrital, fine carbonatic particles, and vegetal debris. This type of facies would have been deposited under a persistent but shallow water column during seasonal flooding and wetter periods (Fig 2B). The other extreme consists of stromatolite-like laminae, and would represent periods of low water level conditions (drier periods) with growth of benthic microbial mats (Fig 2B). A taphonomic analysis comparing the fossil and facies associations indicates that the ‘drier facies’ with microbial mats contain abundant fossils and low richness, whereas the ‘wetter facies’ have fewer fossils but are highly diverse in taxa [40]. The occurrence of mats in the Las Hoyas laminated limestones [46] and in the fossils [51,53] has been crucial to the understanding of the processes and type of preservation in this Konservat-Lagerstätte. Microbial mats are especially relevant as a mechanism to promote the exquisite preservation and abundance of fossils, because they protect carcasses from progressive degradation and induce mineral precipitation, leading to the formation of lithified layers [54–56]. The mechanisms involved in such preservation have been experimentally tested on microbial mat communities growing in tanks under controlled conditions and using different animal carcasses. These essays have verified that mats clearly prevent skeletal disarticulation, retard decay, promote biomineralization of the organic remains, and induce the formation of moulds and replicas [54–56].

## The Las Hoyas ichnoassemblage

The ichnoassemblage of Las Hoyas as previously described is a fine example of the *Mermia* archetype [57], which typifies lacustrine environments [58,59]. The ichnoassemblage includes small, simple, horizontal, shallow-tier invertebrate burrows associated (even in the same layer) with fish-trails and tetrapod trackways [60]. These ichnofossils were described as mostly produced by aquatic organisms (i.e., benthic animals such as crustaceans, worms, insect larvae, and fish). Among invertebrate tracks, *Treptichnus pollardi* is the dominant pattern, together with *Cruziana* isp., *Helminthoidichnites tenuis*, *Lockeia* isp., *Palaeophycus tubularis*, and *Planolites montanus*. Fish trace fossils are recorded as the common ichnospecies *Undichna unisulca* [61]. In addition to this aquatic fauna, some layers also contain traces of air-breathing terrestrial tetrapods such as crocodiles and dinosaurs [62,63]. The most significant part of the Las Hoyas ichnoassemblage pending description are the coprolites, which are the aim of the present contribution.

## Taphonomic characterization

### Relative abundance of associations

The systematic excavations carried out at the locality involved *in situ* taphonomic and sedimentary data retrieval (Fig 1D). Data have been collected during annual excavations since 1985. Coprolites are one of the most abundant fossil remains from the Las Hoyas fossil record. The systematic layer-by-layer sampling of the Las Hoyas successive beds allows for comparison between the abundance of coprolites and body fossils such as bivalves, crustaceans, insects, and fishes (Table 1 in [40], [46]). The data presented (Fig 2) correspond to five 25 to 30 m^2^ sampling squares: PS96, PS98, LBS, UBS, and LmBS [40]. The successive layers excavated at these five squares include drier and wetter facies [40] (Fig 2A). The number of coprolites on each layer ranges from 1 specimen in 30 m^2^ to 177 specimens in 60 m^2^. The highest coprolite abundance, 96%, is associated with the drier facies (Fig 2B). Coprolites represent 26% of the fossil association in the drier facies, whereas they represent only 13% of the fossil association in the wetter facies (Fig 2C).

### Taphonomic features and elemental composition

**Fig 3:**
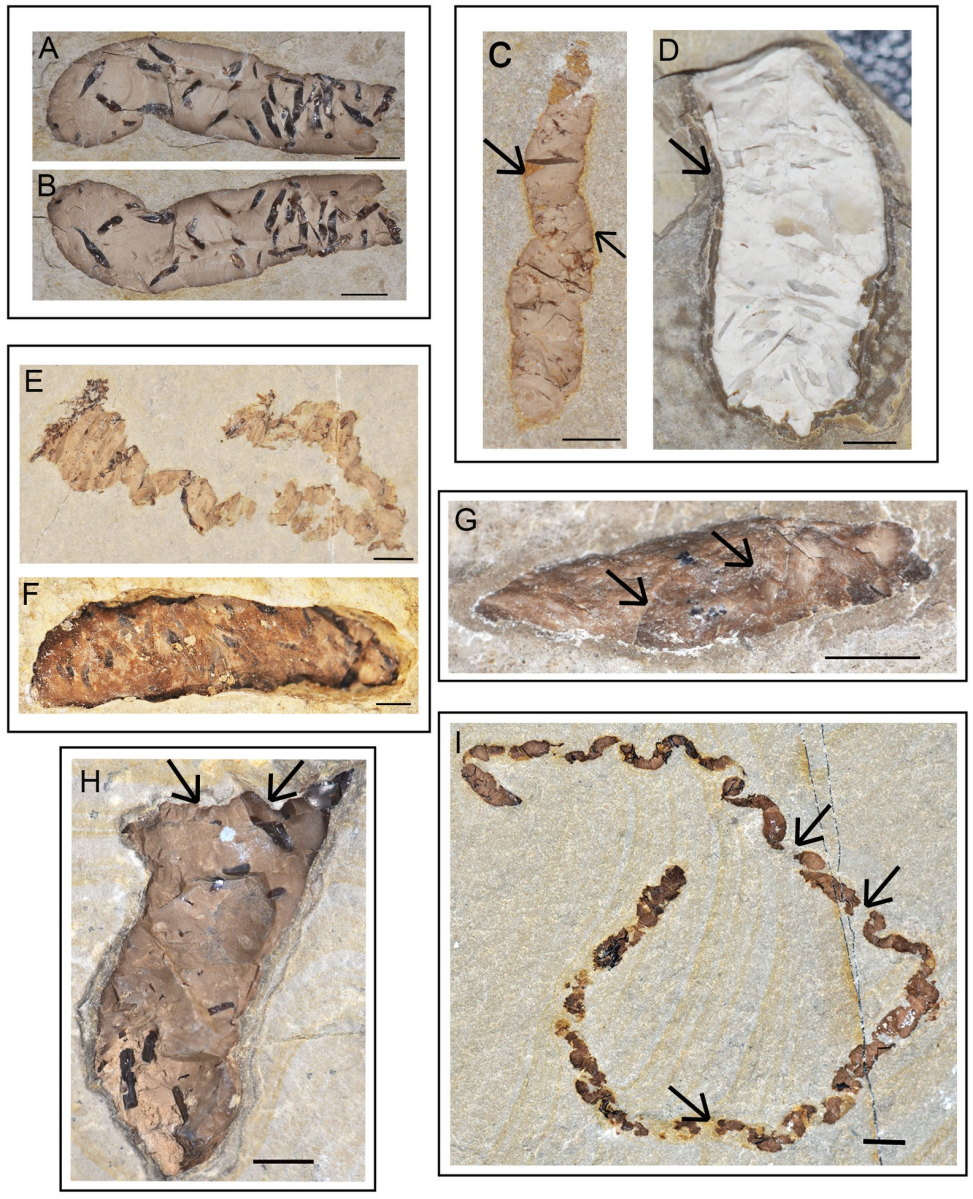
Taphonomic features from Las Hoyas coprolites. **A-B**. Part and counter- part slabs; specimens MCCM-LH21067a and MCCM-LH21067b respectively. **C**. Ferruginous reddish crust as pointed by arrows; specimen MCCM-LH-GQ15-006b. **D**. Nodular laminated envelope as pointed by arrows; specimen MCCM-LH-LI15-001a. **E**. Less dense coprolites flat on the bedding plane; specimen MCCM-LH8036a. **F**. Thicker coprolites are partially embedded into several bedding planes; specimen MCCM-LH21147. **G**. Thin wrinkles in dorsal view as pointed by arrows; specimen MCCM-LH23035. **H**. Breakage of a coprolite end producing an irregular or peaked edge (pointed by arrows); specimen MCCM-LH16609b. **I**. A long coprolite whose continuity throughout its longitudinal dimension is interrupted (interruptions pointed by arrows); specimen MCCM-LH21382b. Scale bars: 2 mm.

The Las Hoyas coprolites are preserved mostly as part and counter-part (Fig 3A and 3B). Each slab is distinguished by the letter ‘a’ and ‘b’ in the specimen number. The taphonomic features studied follow the criteria used in [23], they correspond to: (1) contact with the sediment, (2) desiccation and surface marks, (3) breakage and (4) abrasion (Fig 3). The general taphonomic patterns of the Las Hoyas coprolites are:

1. They are enclosed with their long axis parallel to the lamination of the limestones. Most of them are three-dimensionally preserved with a strong relief. There is no apparent difference between the sediment of the host rock and the sediment that envelopes the coprolite. Some coprolites have a ferruginous reddish crust (Fig 3C), and a few coprolites preserve a nodular laminated envelope (Fig 3D). Less dense coprolites lay on the bedding plane (Fig 3E), whereas thicker coprolites are embedded into several bedding planes (Fig 3F).
2. When the coprolite directly exposes its surface in dorsal view, no deep marks are observed, but thin occasional wrinkles are present (Fig 3G). Neither external nor internal deep desiccation cracks are detected.
3. The completeness of the coprolites is high, maintaining their original contour and shape as deposited in the substrate. Around 20% of the studied specimens show breakage at the coprolite end, producing irregular or peaked edges (Fig 3H). Some long coprolites may interrupt their continuity throughout their longitudinal dimension (Fig 3I).
4. Coprolites do not show any signs of abrasion, and maintain pinched edges, coprolite matrix volume, and contours with angular borders.

**Fig 4:**
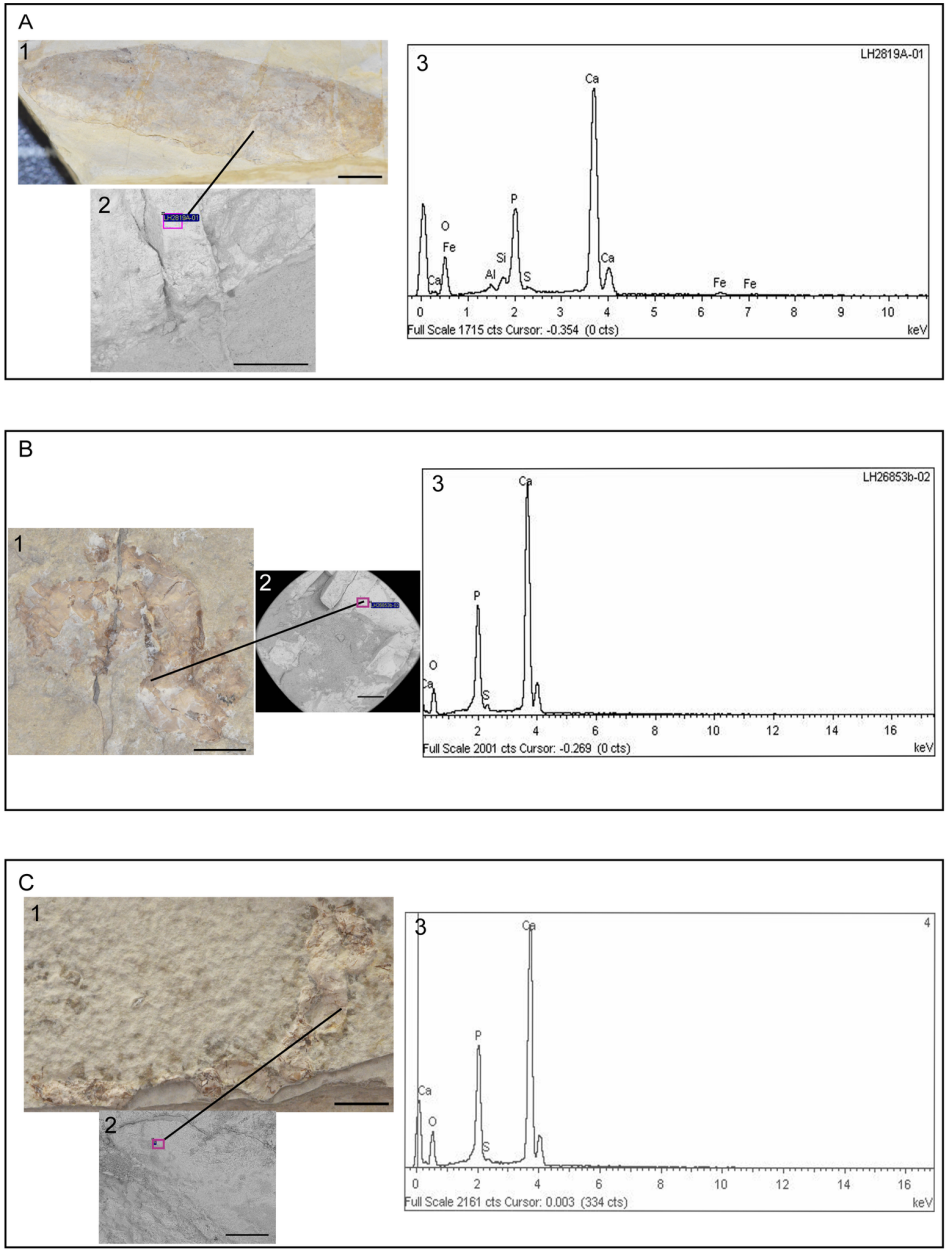
SEM analyses on coprolites. **A.** Specimen MCCM-LH28719a; **B.** Specimen MCCM-LH26853b. **C.** Specimen MCCM-LH35393. In each case **(1)** corresponds to the analysed coprolite. Scale bar: 5 mm; **(2)** SEM image (Secondary Electrons): the violet label indicates the area where the analysis was carried out. Scale bar: 1 mm (except in C, whose scale bar is 200μm), and **(3)** X-ray spectra of the coprolite.

The elemental composition of the coprolite matrix in 17 specimens was determined at the Museo Nacional de Ciencias Naturales (MNCN) in Madrid by energy-dispersive X-ray analyses (EDX analyses) with a low vacuum Environmental Scanning Electron Microscope (ESEM_QUANTA200). These analyses revealed that the main elements of the coprolite matrix are oxygen, phosphorous and calcium, with average percentages of 45–55% for oxygen, 9–15% for phosphorous, and 40–50% for calcium. Some trace elements such as aluminium, silica, sulphur, and iron were also detected. The main elements of their corresponding host rock (the laminated limestones in which coprolites are embedded) are oxygen (43–53%) and calcium (38–51%), with aluminium, silica, carbon, phosphorous, iron, and manganese as minor and trace elements (Fig 4 and Table 1).

**Table 1:** EDX analyses for coprolites and their corresponding host rock.

Wt%	P	Ca	O	Al	Si	S	Fe
**LH28719a (coprolite)**	11.3	39.8	44.9	0.7	1.4	0.7	1.2
**LH28719a (host rock)**	-	38.4	52.9	1.4	2.4	-	0.8
**LH26853b (coprolite)**	14.4	45.7	39.0	-	-	0.8	-
**LH26853b (host rock)**	-	55.0	43.8	0.4	0.8	-	-
**LH35393 (coprolite)**	14.4	43.2	41.9	-	-	0.5	-
**LH35393 (host rock)**	0.5	50.5	48.2	0.4	-	0.4	-

## Morphological characterization

The coprolites studied for the present paper are housed at the Museo de las Ciencias de Castilla-La Mancha (MCCM) in Cuenca, Spain, where they are part of the Las Hoyas (LH) collection. The coprolite collection contains approximately 2,000 specimens. Following a preliminary overall observation, we selected a subsample of 433 specimens for this study (S1 Table), in a manner as to guarantee the most significant representation of the disparity observed in the whole coprolite collection. These 433 specimens were examined using non-destructive techniques. They were photographed at a macro-scale in order to note details of their morphology, overall shape, coprolite matrix colour, and large inclusions by using a D5100 Nikon reflex Camera and an OLYMPUS SZX16 light microscope at the Biology Department of Universidad Autónoma de Madrid (UAM).

The observations of the Las Hoyas coprolites revealed a remarkable morphological diversity. In this section we provide a comprehensive description and distinction of different morphotypes. The primary features applied on the categorization of the coprolite disparity are overall shape and outline. In addition, each morphotype is characterized by a variety of secondary features such as size, shape of the ends, coprolite matrix colour, and comparative density of inclusions and coprolite matrix. All these variables appear in multiple combinations, characterizing the overall morphology of each particular morphological type. The overall shape of the coprolite encompasses the form and geometry of the faecal mass ensemble by including features related to its continuity or pliability, as well as its surface. The outlines can be straight, sinuous, or curved. Observations revealed that the comparative nature of the coprolite ends (i.e., similar or clearly different ends) is an important characteristic of each particular type that is useful for an effective identification. The coprolite matrix colour was categorized into three states: light (whitish), medium (light-brownish), or dark (dark-brownish). The comparative density of inclusions and coprolite matrix allowed their distinction into four categories: (1) coprolites with their matrix entirely visible, with very scarce or no inclusions; (2) coprolites containing inclusions cover about 1/3 of the observed matrix surface; (3) coprolites containing inclusions cover about 2/3 of the observed matrix surface; (4) coprolites whose matrix is occupied almost entirely by inclusions. Diameter and length are given in millimetres (mm): length was measured in the inferred direction of extrusion, and diameter was taken perpendicular to the inferred direction of the extrusion.

**Fig 5:**
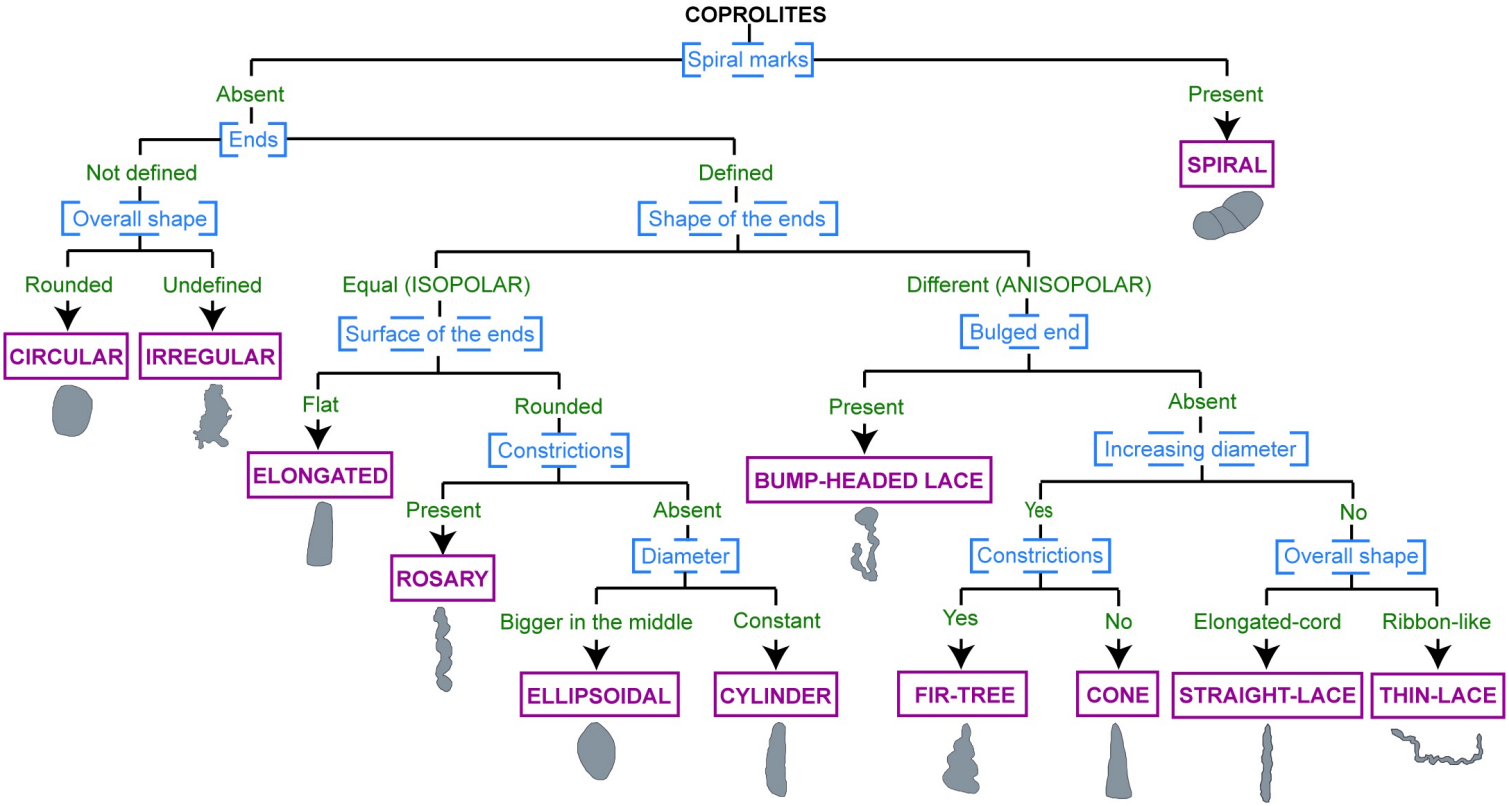
Dichotomous key as proposed for the coprolites from Las Hoyas. Blue letters and dashed lines represent the characters. Green letters and no lines represent alternative character states. Pink letters and continuous lines represent the different morphotypes proposed for the Las Hoyas coprolite disparity.

In order to present this complexity in a comprehensive and organized manner, we combined these characters and constructed a dichotomous key that provides a path for the categorization and diagnosis of the different morphotypes. This key will help to account for the range of coprolite diversity at Las Hoyas, and can provide the basis to categorize any additional specimens or new morphotypes (Fig 5).

### Dichotomous key

The morphological key was constructed by observing the 433 specimens of the selected subsample, so that the variation in each of the features was tested. Only eight out of the 433 specimens differ slightly from any combination of characters as provided by the dichotomous key, probably due to poor preservation. Some of the features consistently observed (including size, coprolite matrix colour, and comparative density of inclusions and coprolite matrix) were not useful in order to differentiate the coprolite types. Such features were not morphotype-specific, they were therefore omitted from the construction of the key, but used for the corresponding complete descriptions (see below). In contrast, the overall shape and the surface of the ends proved to be very useful characteristics for the construction of the dichotomous key.

The first branch in the dichotomous key discerns between spiral and non-spiral coprolites. Non-spiral coprolites may be grouped into defined or no defined ends. The overall shape of the coprolites can be rounded, undefined, elongated-cord, or ribbon-like. The shape of the ends can be equal or different. We use here the terms proposed by Thulborn [3], who describes coprolites as isopolar when they have similarly shaped ends, and anisopolar when the shape of each end is different.

Other characters necessary to account for the range of morphological disparity are: surface of the ends (isopolar coprolites) being either flat or rounded; presence or absence of constrictions, regardless of the overall shape; ‘diameter’ refers to the diameter being roughly similar throughout the entire length or expanded in the middle of the coprolite. Regarding the shape of the coprolite ends, the characteristic ‘bulged end’ refers to the presence of a large lump in only one of the ends. The characteristic ‘increasing diameter’ refers to a continuous increase in the diameter of the coprolite from the minimum diameter at one end to the maximum diameter at the other end.

### Morphotypes

No standard pattern or method for morphological categorization of coprolites (for instance, [64]) has been generally followed so far. On the contrary, authors have traditionally provided independent, ad hoc morphological categorizations of their own in terms of the preservation of the material, number of specimens available, and depositional environment where coprolites were encountered. Because there is no standardized method, we have applied previously used characteristics, as well as some new features, necessary to account for the morphological disparity of the Las Hoyas coprolites. Twelve distinguishable morphotypes are herein described on the basis of the features just presented above. The assessment of their potential producers is usually an untestable hypothesis, so it is considered an irrelevant, potentially misleading matter for the strict morphologic characterization of each morphotype, so such possible assessments are just roughly discussed in Section 6.2 below. S2 Table is a comparative synthesis of the descriptions in the text below, with an appreciation of analogous coprolite shapes as presented by [64].

**Fig 6:**
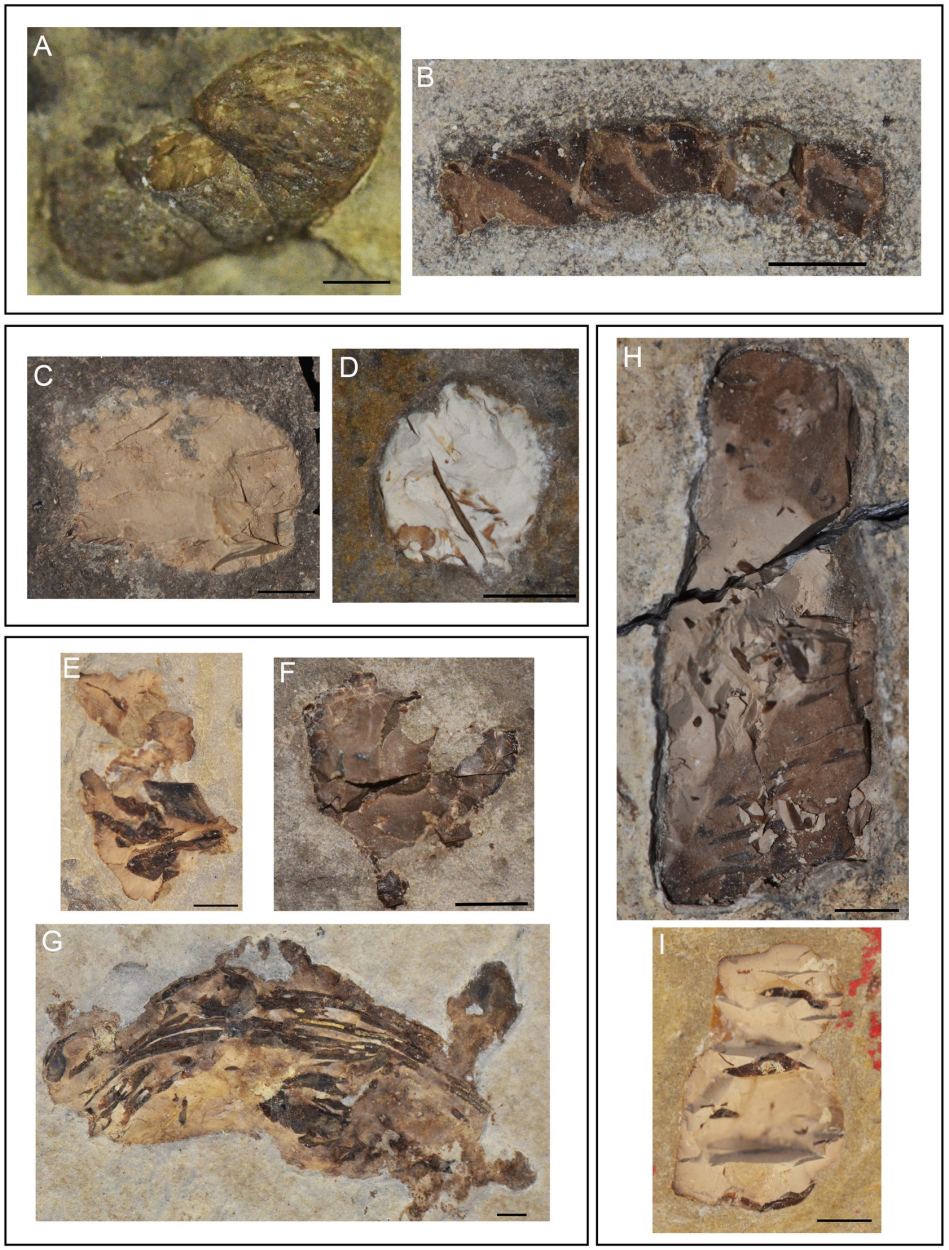
Examples of coprolite morphotypes from Las Hoyas. **A-B**. Spiral: specimens MCCM-LH22349 and MCCM-LH-LI15-032a, respectively. **C-D**. Circular: specimens MCCM-LH-LI15-012 and MCCM-LH21425a. **E-G**. Irregular: specimens MCCM-LH8192, MCCM-LH-LI15-006, and MCCM-LH27015a. **H-I**. Elongated: specimens MCCM-LH21056 and MCCM-LH16516a1. Scale bars: 2 mm.

**Spiral (Fig 6A and 6B)**
**General description:** With spiral marks, in reference to oblique strips on the surface of the coprolite, which vary in number. The width of the strips is regular; strips may occupy the entire coprolite.
**Overall shape:** Elongated.
**Outline:** Straight.
**Diameter at mid-length:** 2–6 mm.
**Length:** 9–12 mm.
**End shapes:** Similar shape within individual coprolites but varies among different spiral coprolites.
**Coprolite matrix colour:** Medium or dark.
**Density of inclusions:** Category 1.
**Kind of inclusions:** Some specimens with no visible inclusions, other specimens with small inclusions impossible to identify.

**Circular (Fig 6C and 6D)**
**General description:** They are rather flat, lentil-like, without relevant volume (no spheroidal or disc-shaped). They are mostly ‘imperfect’ circles with roughly rounded outline.
**Overall shape:** Rounded.
**Outline:** Curved and irregular.
**Diameter at mid-length:** 7.5–100 mm.
**Length:** Not applicable.
**End shapes:** No defined ends.
**Coprolite matrix colour:** Light.
**Density of inclusions:** Category 1 to 2.
**Kind of inclusions:** Some remains of bones (fish vertebrae and scales).

**Irregular (Fig 6E–6G)**
**General description:** This assembly includes all coprolites that have not been grouped as a defined morphotype, including those presenting circumvolutions, an unclear major dimension, and/or a mosaic of shapes.
**Overall shape:** Undefined.
**Outline:** A variety of possible combinations.
**Diameter at mid-length:** (if applicable) 2–39 mm.
**Length:** 6–82 mm.
**End shapes:** Not applicable.
**Coprolite matrix colour:** Light or dark.
**Density of inclusions:** Category 1 to 3 (differing among specimens).
**Kind of inclusions:** Different on each particular specimen: no remains, some plant remains, arthropod remains, and/or fish scales.

**Elongated (Fig 6H and 6I)**
**General description:** They show a straight longitudinal axis with flat ends, the ensemble conferring a roughly rectangular shape. They do not have noticeable volume and are usually flat.
**Overall shape:** Rectangular.
**Outline:** Straight.
**Diameter at mid-length:** 2–23 mm.
**Length:** 6–29 mm.
**End shapes:** Isopolar, flat ends.
**Coprolite matrix colour:** Light, medium or dark (tends to be light-medium colour).
**Density of inclusions:** Category 1 to 4 (differing among specimens).
**Kind of inclusions:** Fish scales and thin bony remains.

**Fig 7:**
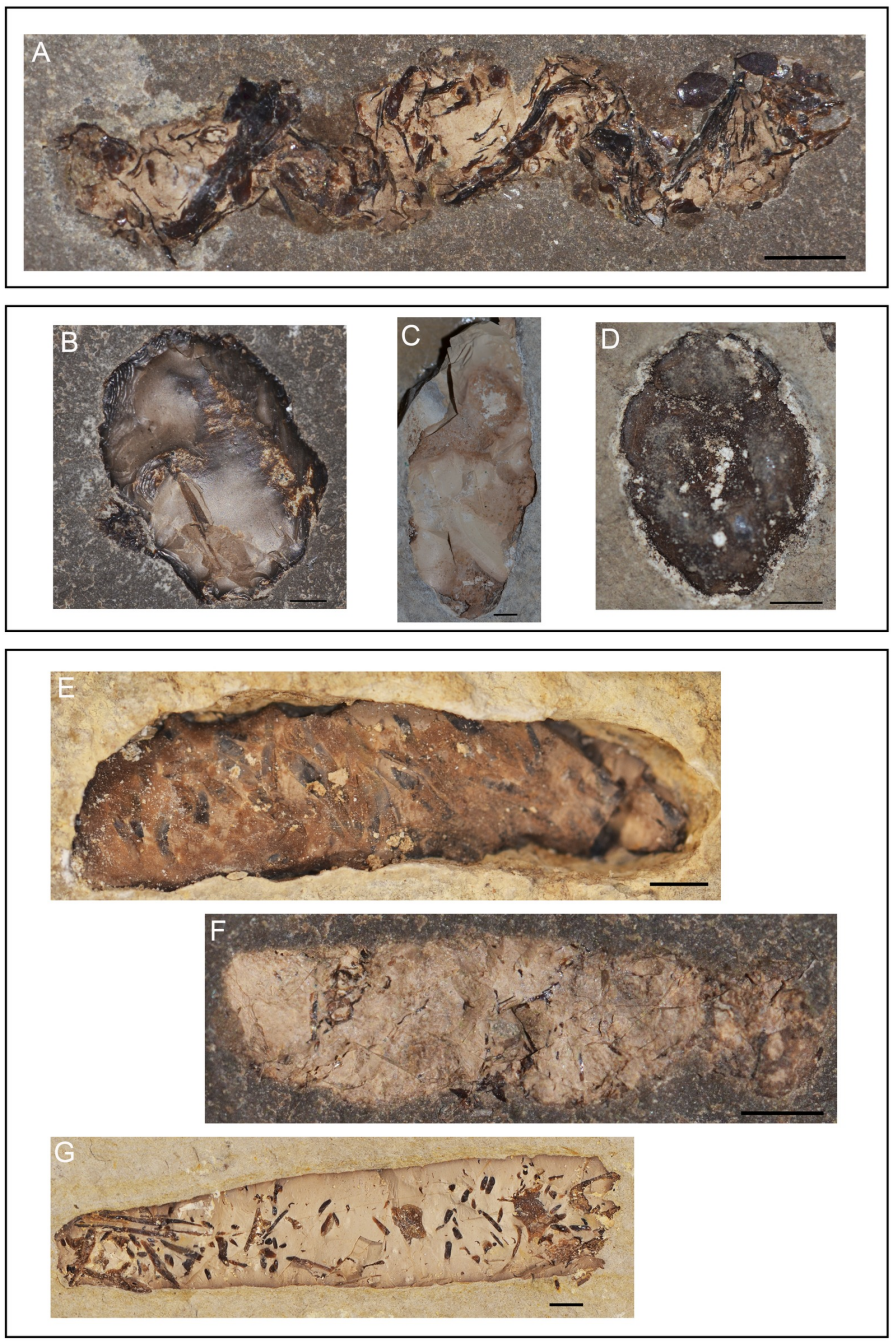
Examples of coprolite morphotypes from Las Hoyas. **A**. Rosary-like: specimen MCCM-LH15505a. **B-D**. Ellipsoidal: specimens MCCM-LH21160a, MCCM-LH-8172(11) and MCCM-LH-GQ15-002, respectively. **E-G**. Cylinder: specimens MCCM-LH21147, MCCM-LI15-019, and MCCM-LH21244b. Scale bars: 2 mm.

**Rosary (Fig 7A)**
**General description:** They present constrictions in the coprolite matrix throughout the longitudinal axis. These constrictions separate a series of wide bumps joined by narrow tracts. The number of constrictions is consistently greater than two. It can be suggested that these bumps and constrictions could indicate sphincter contractions during defecation.
**Overall shape:** Segmented into bumps.
**Outline:** Sinuous.
**Diameter at mid-length:** 1–5 mm.
**Length:** 12.5–24 mm.
**End shapes:** Isopolar, rounded.
**Coprolite matrix colour:** Medium.
**Density of inclusions:** Category 1 to 3 (differing among specimens).
**Kind of inclusions:** Thin bony remains and thick elements that could be scales or other bones.

**Ellipsoidal (Fig 7B–7D)**
**General description:** These coprolites show no special elongation, but distinct minor and major axes: proportion between axes is usually 1/2 to 1/3. The diameter is bigger at mid-length. These coprolites are not as flat as those of the circular morphotype.
**Overall shape:** Roughly ovoid.
**Outline:** Straight.
**Diameter at mid-length:** 1.5–32 mm.
**Length:** 4–75 mm.
**End shapes:** Isopolar, rounded.
**Coprolite matrix colour:** Light, medium, or dark.
**Density of inclusions:** Category 1 to 4 (differing among specimens).
**Kind of inclusions:** Depends on the specimen studied: no inclusions, bones, fish scales or vegetal remains.

**Cylinder (Fig 7E–7G)**
**General description:** The width throughout the longitudinal axis of the coprolite is roughly constant. Some specimens may have a comparatively wider diameter and consequently more volume, showing a stout and dense condition.
**Overall shape:** Elongated, with notable volume.
**Outline:** Straight to slightly curved.
**Diameter at mid-length:** 1.5–20 mm.
**Length:** 8–100 mm.
**End shapes:** Isopolar, rounded.
**Coprolite matrix colour:** Light, medium, or dark.
**Density of inclusions:** Category 1 to 3 (differing among specimens).
**Kind of inclusions:** Thread-like bony elements (maybe scales embedded perpendicularly in the coprolite matrix) and thick bones.

**Fig 8:**
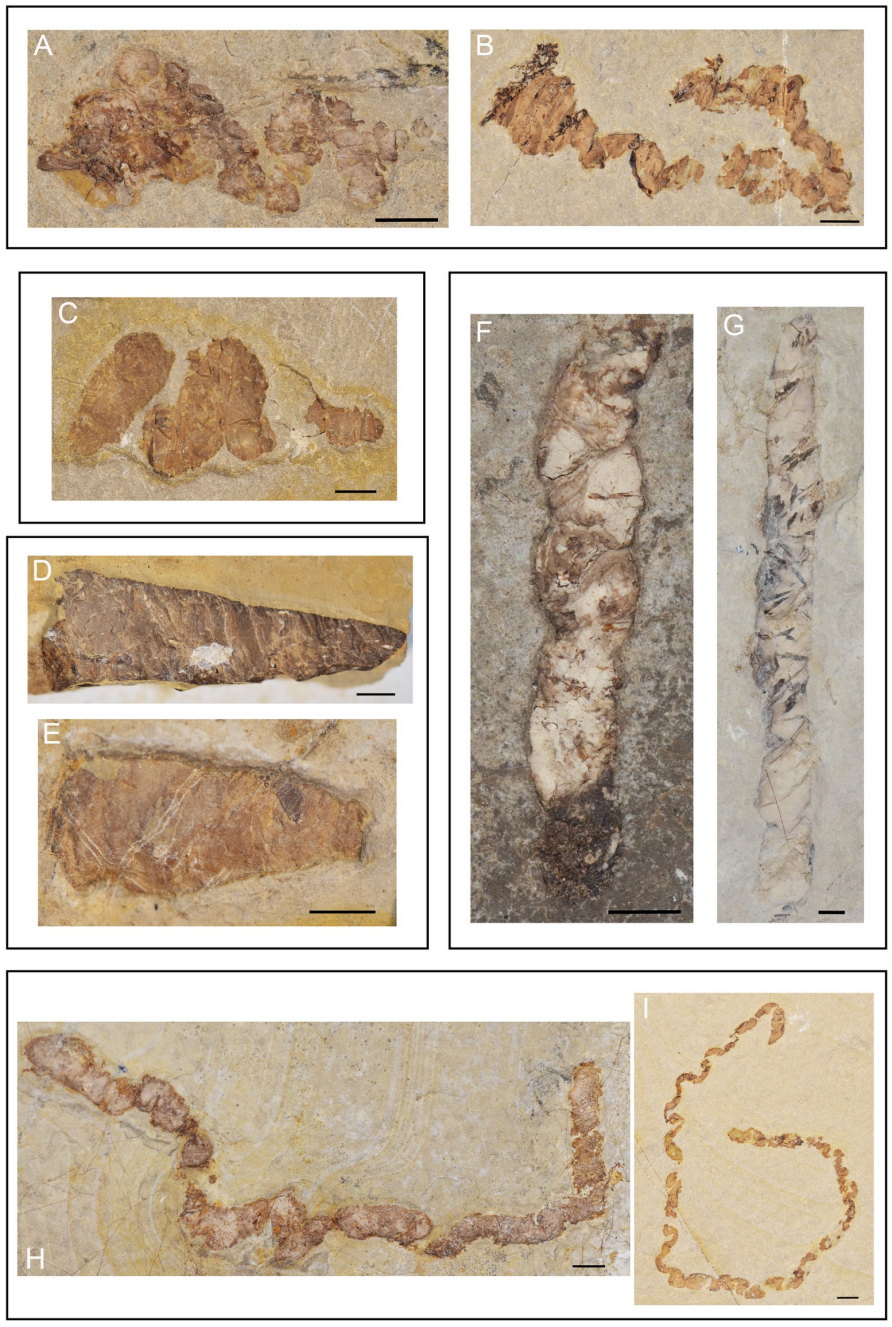
Examples of coprolite morphotypes from Las Hoyas. **A-B**. Bump-headed lace: specimens MCCM-LH21202 and MCCM-LH8036a, respectively. **C**. Fir-tree-like: specimen MCCM-LH21358a. **D-E**. Cone-like: specimens MCCM-LH21192a and MCCM-LH16602a. **F-G**. Straight lace: specimens MCCM-LH-LI15-015 and MCCM-LH-LI15-005. **H-I**. Thin lace: specimens MCCM-LH-LI15-016 and MCCM-LH21382a. Scale bars: 2 mm.

**Bump-headed lace (Fig 8A and 8B)**
**General description:** In reference to one of the ends, which shows a big bulge in comparison with the other end. The bulge is at least twice as wide as the rest of the coprolite.
**Overall shape:** Elongated cord with a distinct bulge.
**Outline:** Sinuous.
**Diameter at mid-length:** 0.5–5 mm.
**Length:** 8–46 mm.
**End shapes:** Anisopolar, large bulge at one end.
**Coprolite matrix colour:** Light, medium, or dark.
**Density of inclusions:** Category 2 to 4 (differing among specimens).
**Kind of inclusions:** Thread-like bony elements (probably scales embedded perpendicularly in the coprolite matrix) and rings (probably tiny fish vertebrae embedded in the coprolite matrix).

**Fir-tree-like (Fig 8C)**
**General description:** In reference to the sequence of ‘bumps’ that decrease progressively from a wide to a very narrow end. There are two to four constrictions separating those bumps.
**Overall shape:** Triangular.
**Outline:** Sinuous (more or less regular).
**Diameter at mid-length:** 1–15 mm.
**Length:** 12–27 mm.
**End shapes:** Anisopolar, the smaller end is rounded and the other is almost straight or slightly bent.
**Coprolite matrix colour:** Light or medium.
**Density of inclusions:** Category 1 to 4 (differing among specimens).
**Kind of inclusions:** Thin bony remains, big bones and scales.

**Cone (Fig 8D and 8E)**
**General description:** The main character is that the diameter increases throughout the longitudinal axis, without constrictions. Their length is at least twice their width. One of the ends coincides with the maximum diameter of the coprolite, the other end with the minimum diameter.
**Overall shape:** Cone to tear-drop.
**Outline:** Straight.
**Diameter at mid-length:** 1.5–15 mm.
**Length:** 3–24 mm.
**End shapes:** Anisopolar, the smaller end can be sharp or a bit rounded, and the other one is almost straight or slightly bent.
**Coprolite matrix colour:** Light, medium, or dark.
**Density of inclusions:** Category 1 to 3 (differing among specimens).
**Kind of inclusions:** Bones, fish scales, some possible ostracods.

**Straight lace (Fig 8F and 8G)**
**General description:** Longitudinal axis long and straight, unfolded, with a roughly similar diameter throughout its length. Length can be 4–10 times the corresponding diameter.
**Overall shape:** Elongated cord.
**Outline:** Sinuous.
**Diameter at mid-length:** 1.5–4 mm.
**Length:** 12–55 mm.
**End shapes:** Anisopolar, one end always rounded and the other end flat to sharp.
**Coprolite matrix colour:** Light, medium, or dark.
**Density of inclusions:** Category 2 to 4 (differing among specimens).
**Kind of inclusions:** The most abundant remains are thick bones, scales, and thin bony fragments.

**Thin lace (Fig 8H and 8I)**
**General description:** Folded onto themselves, as their length is 10 times their width (or more). They have a roughly identical diameter throughout their length.
**Overall shape:** Ribbon-like.
**Outline:** Sinuous.
**Diameter at mid-length:** 1–4 mm; there is a single specimen whose diameter is 14 mm.
**Length:** 12–90 mm; same specimen just mentioned is 150 mm long
**End shapes:** Anisopolar, one end is sharp and the other one is flat or rounded.
**Coprolite matrix colour:** Light, medium, or dark.
**Density of inclusions:** Category 2 to 4 (differing among specimens). In coprolites with very abundant remains it is virtually impossible to observe the coprolite matrix due to the concentration of inclusions
**Kind of inclusions:** Thread-like structures (probably scales embedded in coprolite matrix), rings (probably tiny fish vertebrae embedded in coprolite matrix) and thin bony remains (some of them seem to be segmented fin rays).

### Morphotype abundance

**Fig 9:**
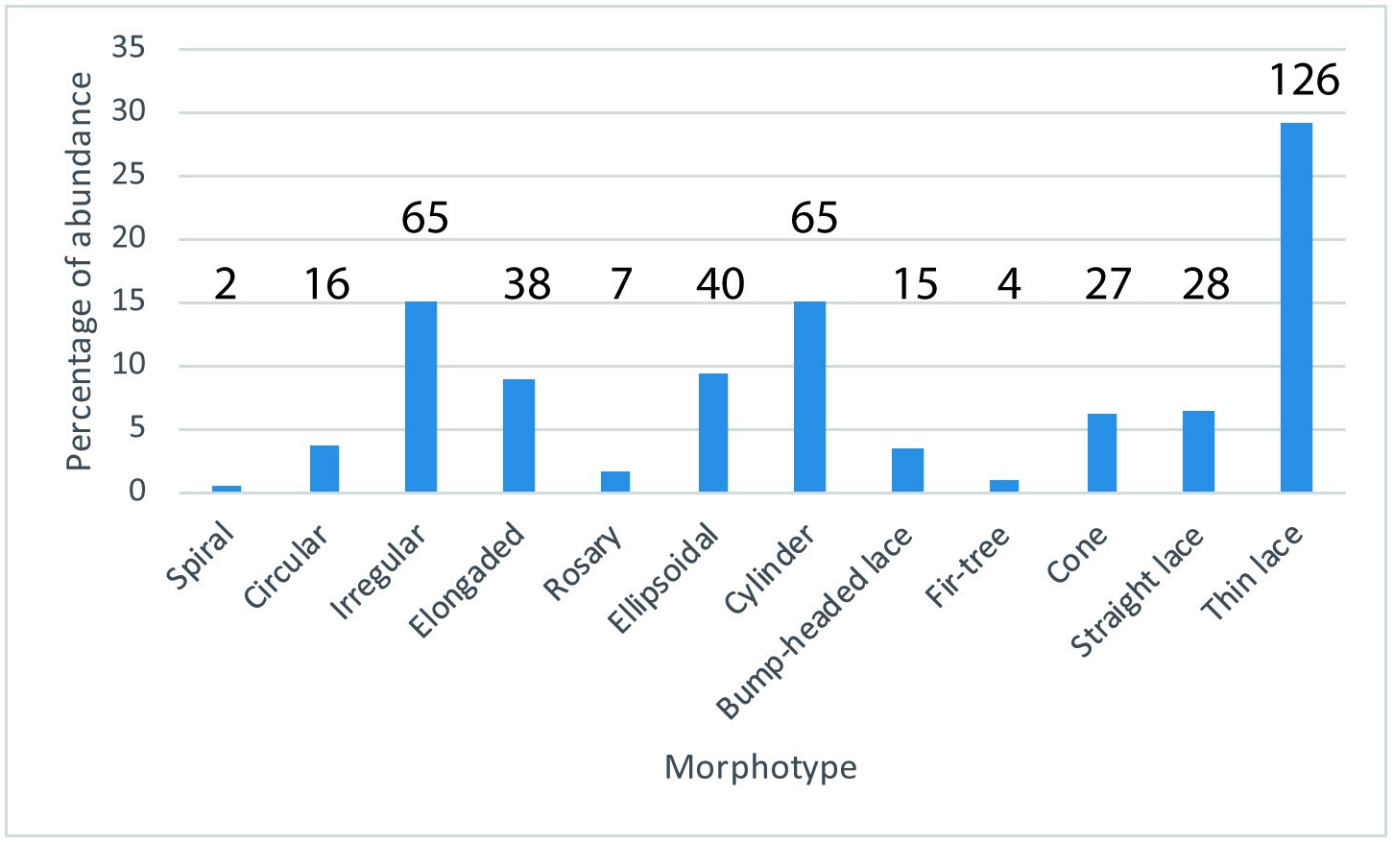
Relative abundance of the morphotypes. The number above each bar is the absolute number of coprolites for the corresponding morphotype (total N = 433).

The abundance, both numerical and relative, of each particular morphotype is presented in Fig 9 (N = 433). The sample selection contains two sources of bias: field collection and museum selection. The percentages obtained are consistent with our practical field experience; the most abundant morphotype, considerably, is thin lace (29%), followed by cylinder (15%), irregular (15%), and ellipsoidal (9%). The least abundant morphotypes are spiral (0.5%), fir-tree-like (0.9%), and rosary (1.5%).

### Coprolite size according to morphotype

**Fig 10:**
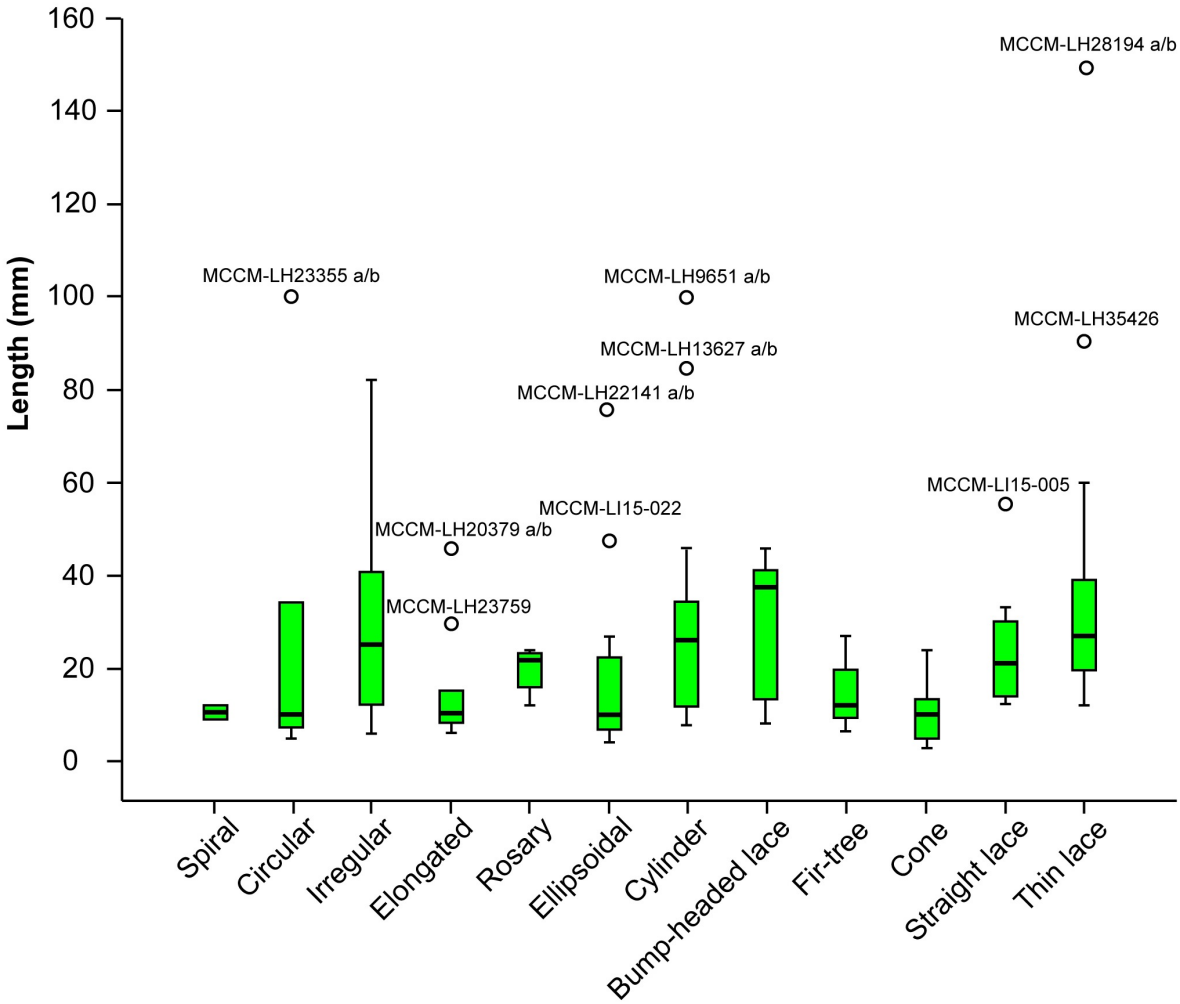
Coprolite lengths for each particular morphotype. Box-plot graphic representing length values for specimens of the different morphotypes (N = 141). Circles represent the outliers for the corresponding morphotype, box represents the quartiles, thin line represents the median, and whiskers represent interquartile range x 1,5 (IR x 1,5).

There is a considerable overlap in the morphotype size ranges. Circular, ellipsoidal and elongated are skewed towards smaller sizes, whereas cylinder, rosary and bump-headed lace are skewed toward larger sizes (note the position of the median lines within the quartile boxes in Fig 10). The most common size range for the Las Hoyas coprolites is from 10 to 40 mm (Fig 10). Only three coprolite specimens from Las Hoyas measure 5 mm long or less, and they belong to three different morphotypes each: circular, ellipsoidal, and cone.

## Discussion

The term *coprolite assemblage* has been defined as groups of icnhofossils preserved in a rock unit and depositional environment [65]. In order to refine this concept, we have applied the general definitions of *assemblage* and *association* to the coprolites [66]; i.e. an *assemblage* is any ensemble of fossils that is recovered from the same layer, and an *association* is the combination of the fossils themselves plus their taphonomic properties and lithology. Therefore, we use the term *coprolite association* or *coproassociation* for any coprolite assemblage that integrates all the biological, taphonomic, and lithologic properties into their fossil record. These properties depend directly on the structure and conditions of the original environment, therefore providing information about the palaeoecosystem.

### Environmental properties

Overall, the Las Hoyas coprolite record is a clear example of a lentic freshwater association characterized by its exquisite preservation and a variety of shape and size. The Las Hoyas coproassociation adds new information to the trace-fossil assemblages previously reported as *Mermia ichnofacies* from several lacustrine Barremian localities [58]. The Las Hoyas coproassociation is dominated by thin-lace and cylinder morphotypes containing a profuse abundance of inclusions in the coprolite matrix. The Las Hoyas coprolites form a continuum in size (as measured by the corresponding major dimension): the smaller size is rather tiny (5 to 8 mm). Such small size has been previously reported from some lacustrine environments [23,38,39].

The preservational features of fossil coprolites are congruent with those exhibited by faeces deposited in aquatic environment. According to the biostratinomic observations in [5,20,21], deposition in an aquatic environment is indicated by the absence of desiccation marks and flattened surfaces on the coprolites. Faeces dropped in dry environments become dehydrated, and after prolonged aerial exposure, their volume, shape, and size are often altered [3]. Such indications of aerial exposure are absent in most of the Hoyas coprolites, indicating absence of transport in a subaerial environment (Fig 3).

Other taphonomic features of the Las Hoyas coprolites confirm they are autochthonous. Lack of transport is indicated by absence of abrasion even on the smallest and narrowest specimens. Transport of aquatic faeces is common in rivers, resulting in dense allochthonous accumulations in small areas of dead-water zones, backwaters, river margins, and lowlands [39,67]. Such density is very rare in Las Hoyas.

The Las Hoyas coprolites are also interpreted as demic (produced where the animal lived). Vertical transport in the water column is very limited, as indicated by their integrity. A vertical flux is common in recent lakes [67]: the sinking rate of faeces in water is often rapid, and varies with size and faecal compaction [67–69]. In any case, vertical transport in water prompts the fragmentation of the faecal mass [69]. After that, shape is maintained once on the bottom because faeces are bound together with mucus, which is present in aquatic vertebrate and in most invertebrate faeces [67]: the bounded faecal mass may persist in the sediment for weeks [70].

A rapid burial and lithification is crucial to ensure coprolite shape and integrity [20,27,29]. The action of the microbial mats favours both circumstances [40]. In fact, most of the laminated sediments of Las Hoyas are a carbonatic biogenic production of the microbial mats. Mats have been profusely documented at Las Hoyas by taphonomic evidences such as calcified extra polymeric substances (EPS) and different bacterial remains and impressions on bones and soft tissues [51–53]. Despite the lack of direct evidence of coccoid bacteria in the coprolites themselves, some of them show a laminated envelope around the coprolite matrix (Fig 3D) probably caused by mat growth over the faecal mass. We interpret that most coprolites were trapped by growing mats (see Section 2 and references therein for details).

The maximum abundance of coprolites in Las Hoyas occurs in the ‘drier’ facies (see Section 4.1 and Fig 2B). In the Las Hoyas palaeoecosystem microbial mats growth is related to seasonality, as in Recent analogous wetlands such as the Everglades in Florida [71,72]. The maximum periphyton growth occurs during the regeneration stage between the driest and the wettest periods, when the growing mats drift in the water column. This particular stage would have been the most propitious to trap the faeces of the aquatic animals that dwelled in the Las Hoyas waters.

### Biological properties

The properties of the faeces produced by aquatic animals vary with the functional feeding groups of the corresponding organisms: (1) predators, that eat other animals, (2) shredders, that feed on live or dead plant material, (3) scrapers, that feed on biofilms and remove the organic covering on surfaces, and (4) collectors, that are suspension feeders and deposit feeders. In general, the faeces of shredders, scrapers, and collectors are more abundant, but smaller, than those produced by predators [67].

The coprolites from Las Hoyas show features that fall mostly within the predator feeding strategy. Las Hoyas coprolites are usually over 5 mm, which is the common size range of predator vertebrate coprolites [70,73]. EDX analyses on coprolites (Fig 4 and Table 1) indicate that the elemental composition of most of them shows high levels of phosphorous and calcium, which suggests a predominant calcium-phosphate composition, typical of carnivorous scats [27]. Furthermore, bones are the most frequent inclusions in Las Hoyas coprolites: the presence of bony tissues facilitates phosphatization, which, in turn, favours coprolite preservation [3]. Most of the fragments observed as inclusions in the Las Hoyas coprolites are fish scale fragments and tiny fish bones (probably vertebrae, fin rays, and ribs), indicating a predominant ichthyophagous diet of the corresponding coprolite producer. However, the precise producer taxon cannot be precisely assessed with this evidence. Modern aquatic macropredator insects include notonectids that can prey on insects, small fishes, and tadpoles [74]. The belostomatid *Iberonepa* recorded at Las Hoyas is an ecological analog of the living notonectids. Other than this, all other possible ichthyophagous predators from Las Hoyas are vertebrates: a variety of fishes (sharks, amiids, coelacanths), urodelans, crocodiles, even turtles. Taxonomic assignation requires further studies, but these general considerations are consistent with the evidence provided so far by the coprolites themselves when compared with the actual body fossil record from Las Hoyas [74].

Concerning feeding strategies, observation of the inclusions can provide preliminary inferences about the type of digestive processes involved. Significant differences in the amount and features of the inclusions have been detected between the two dominant morphotypes, thin lace and cylinder. The density of inclusions of thin lace coprolites corresponds to categories 2–4 as characterized in Section 5 above, inclusions are never scarce. Their inclusions are the most complete, and they can be safely identified as fish vertebrae (osseous rings) and fin rays (segmented elongated remains). The density of inclusions in cylinder coprolites corresponds to categories 1–3 (remains can be scarce). Their inclusions are more fragmentary and they show surface alterations, which render identification quite uncertain. The thin lace and cylinder morphotypes present distinct features that indicate different digestive processes: the former a digestion with either low acidic content or a short retention time of food [29,75], and the latter with a more effective (high acidic) or longer digestion [76].

The unusual, remarkable morphotype variety of the Las Hoyas coprolites and their corresponding features clearly call for additional studies. By combining morphology, digestive processes, and body fossil record, the only morphotype that can be attributed with some certainty to a particular producer is the spiral one: Spiral coprolites can only be produced by fishes and other animals whose digestive tract contains a spiral valve [77]. Spiral valves are present in cyclostomes and in some bony fishes with the exception of teleosts [20]. Therefore, we interpret the spiral coprolite from Las Hoyas to have been most likely produced by sharks, among the known fossil record from the locality. This is congruent with very similar scarcity of both spiral coprolites and fossil sharks in the Las Hoyas record.

Other than this, a precise assessment of any other producer is problematic, even when combining all the data presented in the present paper. Among the rest of the morphotypes, those presenting abundant fish inclusions are mostly straight and thin lace. Therefore, they could reasonably be linked to an ichthyophagous producer according to the indications of the digestive process. In order to suggest a particular producer, size alone is not a reliable indicator, because the extrapolation of coprolite size in an attempt to relate it with the body size of a precise producer is quite problematic [78]. Some morphotypes include coprolites that are much longer than the most common measurement of the corresponding morphotype (Fig 10), which could suggest a different producer taxon but also a larger producer individual of the same taxon, corresponding to different ontogenetic states. For instance, the size of the amiiformes and the coelacanth specimens at Las Hoyas ranges from very small juveniles to large adults [79] resulting congruent with the disparity of sizes found in these coprolite morphotypes (Fig 10).

The other morphotypes are even more difficult to assess. For instance, cylinder coprolites show a diversity of bony inclusions. Possible predator producers among the fossil record of Las Hoyas include reptiles such as theropods, pterosaurs, and crocodiles. Extant crocodile scats present cylindrical, elongated to conical shape [80]. In addition, crocodiles have a very effective digestive system able to decalcify and dissolve bones. The cylinder morphotype of Las Hoyas are congruent with a crocodilian producer based on the shape, alterations, and scarcity of inclusions. In sum, more studies, including molecular analyses, out of the scope of the present contribution, are necessary prior to presenting a more exhaustive discussion of putative producer assessments.

## Conclusions

Las Hoyas provides a remarkable example of a rich coproassociation that was produced in a lacustrine depositional environment interpreted as an inland seasonal and subtropical wetland. The palaeoecosystem favoured and was influenced by the seasonal growing of microbial mats, which would have contributed to preservation of a broad disparity of coprolite shapes. Twelve morphotypes have been distinguished by a combination of features, and ordered into a dichotomous key. The taphonomic features observed indicate that the coprolites are autochthonous and demic ichnofossils. EDX analyses and the kind of inclusions found in the coprolite matrix indicate that most of them were produced by ichthyophagous vertebrates. The shape of the two-dominant morphotypes (thin lace and cylinder), and the evidence of their digestive process are congruent with a variety of possible vertebrate producers, including different fishes and crocodiles. Only spiral coprolites can be attributed to a specific producer in Las Hoyas (sharks). Future research will need to combine all these data with other sources of information, such as a detailed analysis of the contents and other analytical methods, in order to fully integrate the information provided by the coprolites and their possible producers into the complex trophic network of the Las Hoyas wetland palaeoecosystem.

## Supporting information

S1 Table List of the 433 specimens studied, with their corresponding morphotype. (PDF)

S2 Table Coprolites morphotype features presented as a synthetic comparison of the corresponding description, with an appreciation of analogous coprolite shapes based on [64]. (PDF)
