# Supplementary material for: Exceptional coprolite association from the Early Cretaceous continental Lagerstätte of Las Hoyas, Cuenca, Spain
Source: PLoS One. 2018 May 23;13(5):e0196982. doi: 10.1371/journal.pone.0196982 (PMC5965836; doi:10.1371/journal.pone.0196982)
Supplement: S1 Table — (PDF) [file pone.0196982.s001.pdf]

Specimen number	Morphotype
MCCM-LH8036 a/b	Bump-headed lace
MCCM-LH8048 (4) b	Bump-headed lace
MCCM-LH8048 (5) a/b	Bump-headed lace
MCCM-LH15237	Bump-headed lace
MCCM-LH15840	Bump-headed lace
MCCM-LH15993	Bump-headed lace
MCCM-LH15993 a/b	Bump-headed lace
MCCM-LH20254 a	Bump-headed lace
MCCM-LH21189 a/b	Bump-headed lace
MCCM-LH21202	Bump-headed lace
MCCM-LH21415	Bump-headed lace
MCCM-LH28377 1/2	Bump-headed lace
MCCM-LH35393	Bump-headed lace
MCCM-LH-LI15-031	Bump-headed lace
MCCM-LH-SnG11	Bump-headed lace
MCCM-LH2962	Circular
MCCM-LH2969	Circular
MCCM-LH3798	Circular
MCCM-LH8172 (12)	Circular
MCCM-LH9407	Circular
MCCM-LH15745	Circular
MCCM-LH15774	Circular
MCCM-LH15838	Circular
MCCM-LH20281 a/b	Circular
MCCM-LH21087	Circular
MCCM-LH21425 a/b	Circular
MCCM-LH21491 a/b	Circular
MCCM-LH23355 a/b	Circular
MCCM-LH29332	Circular
MCCM-LH32352 (2)	Circular
MCCM-LH-LI15-012	Circular
MCCM-LH2930	Cone
MCCM-LH8014 (1)	Cone
MCCM-LH8014 (3)	Cone
MCCM-LH8048 (6) b	Cone
MCCM-LH9351	Cone
MCCM-LH9373	Cone
MCCM-LH15053	Cone
MCCM-LH15383	Cone
MCCM-LH15424	Cone
MCCM-LH15711	Cone
MCCM-LH15998	Cone
MCCM-LH16200	Cone
MCCM-LH16250	Cone
MCCM-LH16517 a	Cone
MCCM-LH16553	Cone
MCCM-LH16601 b (1)	Cone
MCCM-LH16602 a	Cone
MCCM-LH16609 2plates	Cone

MCCM-LH21106 a/b	Cone
MCCM-LH21127 b	Cone
MCCM-LH21144 a/b	Cone
MCCM-LH21159	Cone
MCCM-LH21192 a/b	Cone
MCCM-LH21281 a/b	Cone
MCCM-LH21293	Cone
MCCM-LH21404	Cone
MCCM-LH-GQ17-010	Cone
MCCM-LH659	Cylinder
MCCM-ALH1237	Cylinder
MCCM-LH2899	Cylinder
MCCM-LH2961	Cylinder
MCCM-LH2966	Cylinder
MCCM-LH4041	Cylinder
MCCM-LH4043	Cylinder
MCCM-LH8048 (2) a/b	Cylinder
MCCM-LH8065 2b	Cylinder
MCCM-LH8175 b	Cylinder
MCCM-LH9475 a/b	Cylinder
MCCM-LH9651 a/b	Cylinder
MCCM-LH13627 a/b	Cylinder
MCCM-LH14098	Cylinder
MCCM-LH14137	Cylinder
MCCM-LH15451	Cylinder
MCCM-LH15452	Cylinder
MCCM-LH15849	Cylinder
MCCM-LH15857	Cylinder
MCCM-LH15893	Cylinder
MCCM-LH15934	Cylinder
MCCM-LH16038	Cylinder
MCCM-LH16212	Cylinder
MCCM-LH16248	Cylinder
MCCM-LH17025	Cylinder
MCCM-LH17075	Cylinder
MCCM-LH20323 a/b	Cylinder
MCCM-LH20327 a/b	Cylinder
MCCM-LH20331	Cylinder
MCCM-LH21030	Cylinder
MCCM-LH21055	Cylinder
MCCM-LH21060	Cylinder
MCCM-LH21067	Cylinder
MCCM-LH21075	Cylinder
MCCM-LH21079	Cylinder
MCCM-LH21118 a/b	Cylinder
MCCM-LH21147	Cylinder
MCCM-LH21154 a/b	Cylinder
MCCM-LH21156 a/b	Cylinder
MCCM-LH21173	Cylinder
MCCM-LH21205	Cylinder

MCCM-LH21225	Cylinder
MCCM-LH21230	Cylinder
MCCM-LH21244 a/b	Cylinder
MCCM-LH21245	Cylinder
MCCM-LH21257 a/b	Cylinder
MCCM-LH21276	Cylinder
MCCM-LH21298	Cylinder
MCCM-LH21316 a/b	Cylinder
MCCM-LH21330	Cylinder
MCCM-LH21349	Cylinder
MCCM-LH21375	Cylinder
MCCM-LH21486 a/b	Cylinder
MCCM-LH23035	Cylinder
MCCM-LH23381 a/b	Cylinder
MCCM-LH28719 a	Cylinder
MCCM-LH28719 b (2)	Cylinder
MCCM-LH-GQ15-001	Cylinder
MCCM-LH-GQ17-011	Cylinder
MCCM-LH-GQ17-013	Cylinder
MCCM-LH-GQ17-014	Cylinder
MCCM-LH-LI15-019	Cylinder
MCCM-LH-LI15-020	Cylinder
MCCM-LH-LI15-026	Cylinder
MCCM-LH-LI15-036	Cylinder
MCCM-LH2927	Ellipsoidal
MCCM-LH4063 (2)	Ellipsoidal
MCCM-LH8014 (4)	Ellipsoidal
MCCM-LH8074 b	Ellipsoidal
MCCM-LH8172 (11)	Ellipsoidal
MCCM-LH8174 b	Ellipsoidal
MCCM-LH8198 a/b	Ellipsoidal
MCCM-LH9012 a/b	Ellipsoidal
MCCM-LH9339	Ellipsoidal
MCCM-LH9371	Ellipsoidal
MCCM-LH15001	Ellipsoidal
MCCM-LH15246	Ellipsoidal
MCCM-LH15369	Ellipsoidal
MCCM-LH15577	Ellipsoidal
MCCM-LH15728	Ellipsoidal
MCCM-LH15815	Ellipsoidal
MCCM-LH15896	Ellipsoidal
MCCM-LH15898	Ellipsoidal
MCCM-LH15902	Ellipsoidal
MCCM-LH15929	Ellipsoidal
MCCM-LH16516 a/b (1)	Ellipsoidal
MCCM-LH20269	Ellipsoidal
MCCM-LH21096 a/b	Ellipsoidal
MCCM-LH21155	Ellipsoidal
MCCM-LH21160	Ellipsoidal
MCCM-LH21203 a/b	Ellipsoidal

MCCM-LH21277	Ellipsoidal
MCCM-LH21280	Ellipsoidal
MCCM-LH21282	Ellipsoidal
MCCM-LH21352	Ellipsoidal
MCCM-LH21354	Ellipsoidal
MCCM-LH21392	Ellipsoidal
MCCM-LH21424	Ellipsoidal
MCCM-LH22141 a/b	Ellipsoidal
MCCM-LH28719 b (1)	Ellipsoidal
MCCM-LH-GQ15-002	Ellipsoidal
MCCM-LH-LI15-003	Ellipsoidal
MCCM-LH-LI15-022	Ellipsoidal
MCCM-LH-LI15-023	Ellipsoidal
MCCM-LH-LI15-024	Ellipsoidal
MCCM-LH4039	Elongated
MCCM-LH8048 (1)	Elongated
MCCM-LH8048 (3) b	Elongated
MCCM-LH9330 a/b	Elongated
MCCM-LH9332	Elongated
MCCM-LH9343	Elongated
MCCM-LH9386	Elongated
MCCM-LH14136	Elongated
MCCM-LH15313	Elongated
MCCM-LH15415	Elongated
MCCM-LH15579	Elongated
MCCM-LH15713	Elongated
MCCM-LH15763	Elongated
MCCM-LH15802a	Elongated
MCCM-LH15836	Elongated
MCCM-LH15891	Elongated
MCCM-LH15925	Elongated
MCCM-LH15930	Elongated
MCCM-LH15965	Elongated
MCCM-LH15994	Elongated
MCCM-LH16211	Elongated
MCCM-LH16516 a/b (2)	Elongated
MCCM-LH16605	Elongated
MCCM-LH20379 a/b	Elongated
MCCM-LH21056	Elongated
MCCM-LH21097 a/b	Elongated
MCCM-LH21102 a/b	Elongated
MCCM-LH21105 a/b	Elongated
MCCM-LH21145	Elongated
MCCM-LH21168	Elongated
MCCM-LH21275	Elongated
MCCM-LH21351	Elongated
MCCM-LH21369	Elongated
MCCM-LH21492	Elongated
MCCM-LH23759	Elongated
MCCM-LH-LI15-001	Elongated

MCCM-LH-LI15-002	Elongated
MCCM-LH-LI15-014 (2)	Elongated
MCCM-LH15861	Fir-tree
MCCM-LH21358 a/b	Fir-tree
MCCM-LH-LI15-008	Fir-tree
MCCM-LH-LI15-021	Fir-tree
MCCM-LH4172 a/b	Irregular
MCCM-LH4190 a/b	Irregular
MCCM-LH7102 a/b	Irregular
MCCM-LH7202	Irregular
MCCM-LH8014 (2)	Irregular
MCCM-LH8192	Irregular
MCCM-LH9101	Irregular
MCCM-LH9240 a/b	Irregular
MCCM-LH9345	Irregular
MCCM-LH9440	Irregular
MCCM-LH9567	Irregular
MCCM-LH9593 a/b	Irregular
MCCM-LH13193	Irregular
MCCM-LH13195 b	Irregular
MCCM-LH13587 a/b	Irregular
MCCM-LH13756	Irregular
MCCM-LH14036	Irregular
MCCM-LH14164	Irregular
MCCM-LH14221	Irregular
MCCM-LH14305	Irregular
MCCM-LH15282 a/b	Irregular
MCCM-LH15336	Irregular
MCCM-LH15591 a/b	Irregular
MCCM-LH15603	Irregular
MCCM-LH15718	Irregular
MCCM-LH16013 a/b	Irregular
MCCM-LH16216	Irregular
MCCM-LH16232	Irregular
MCCM-LH20124 2plates	Irregular
MCCM-LH20173 a/b	Irregular
MCCM-LH20202 a/b	Irregular
MCCM-LH20562	Irregular
MCCM-LH21053 a/b	Irregular
MCCM-LH21076	Irregular
MCCM-LH21108	Irregular
MCCM-LH21120 a/b	Irregular
MCCM-LH21224	Irregular
MCCM-LH21442	Irregular
MCCM-LH22256 a/b	Irregular
MCCM-LH22387 A 1plate	Irregular
MCCM-LH22388 A 1plate	Irregular
MCCM-LH23004	Irregular
MCCM-LH23388 a/b	Irregular
MCCM-LH23389 a/b	Irregular

MCCM-LH26298 a/b	Irregular
MCCM-LH26853 a/b	Irregular
MCCM-LH27015 a/b	Irregular
MCCM-LH27140 a/b	Irregular
MCCM-LH30069	Irregular
MCCM-LH30492 b	Irregular
MCCM-LH32167 b	Irregular
MCCM-LH32352 (1)	Irregular
MCCM-LH32352 (3)	Irregular
MCCM-LH35004	Irregular
MCCM-LH36056 a/b	Irregular
MCCM-LH-GQ15-004	Irregular
MCCM-LH-GQ15-005	Irregular
MCCM-LH-GQ15-008	Irregular
MCCM-LH-LI15-004	Irregular
MCCM-LH-LI15-006	Irregular
MCCM-LH-LI15-009	Irregular
MCCM-LH-LI15-010	Irregular
MCCM-LH-LI15-011	Irregular
MCCM-LH-LI15-013	Irregular
MCCM-LH-LI15-027	Irregular
MCCM-LH15505	Rosary
MCCM-LH16531	Rosary
MCCM-LH21198 a/b	Rosary
MCCM-LH21328 a/b	Rosary
MCCM-LH21336 a/b	Rosary
MCCM-LH23541 a/b	Rosary
MCCM-LH-LI15-030	Rosary
MCCM-LH22349	Spiral
MCCM-LH-LI15-032	Spiral
MCCM-LH764 a/b	Straight lace
MCCM-LH4140 a/b	Straight lace
MCCM-LH8048 (7) a	Straight lace
MCCM-LH8092 a/b	Straight lace
MCCM-LH9107 a/b	Straight lace
MCCM-LH9314	Straight lace
MCCM-LH9489 a/b	Straight lace
MCCM-LH9575 a/b	Straight lace
MCCM-LH15011	Straight lace
MCCM-LH15053	Straight lace
MCCM-LH15240	Straight lace
MCCM-LH15362	Straight lace
MCCM-LH16603 b/b	Straight lace
MCCM-LH16611 a/b	Straight lace
MCCM-LH20198 b	Straight lace
MCCM-LH20546 a/b	Straight lace
MCCM-LH21151	Straight lace
MCCM-LH21228 a	Straight lace
MCCM-LH21390	Straight lace
MCCM-LH21450	Straight lace

MCCM-LH22309	Straight lace
MCCM-LH23132	Straight lace
MCCM-LH-GQ15-006	Straight lace
MCCM-LH-LI15-005	Straight lace
MCCM-LH-LI15-015	Straight lace
MCCM-LH-LI15-029	Straight lace
MCCM-LH-LI15-033	Straight lace
MCCM-LH-LI15-035	Straight lace
MCCM-LH2898	Thin lace
MCCM-LH2898	Thin lace
MCCM-LH2926	Thin lace
MCCM-LH3031	Thin lace
MCCM-LH3035	Thin lace
MCCM-LH3780	Thin lace
MCCM-LH4057	Thin lace
MCCM-LH4058	Thin lace
MCCM-LH4063 (1)	Thin lace
MCCM-LH7329	Thin lace
MCCM-LH8065 b1/b2	Thin lace
MCCM-LH8133	Thin lace
MCCM-LH9032 a/b	Thin lace
MCCM-LH9040	Thin lace
MCCM-LH9106	Thin lace
MCCM-LH9233 a/b	Thin lace
MCCM-LH9234 a/b	Thin lace
MCCM-LH9239	Thin lace
MCCM-LH9241 a/b	Thin lace
MCCM-LH9254	Thin lace
MCCM-LH9301	Thin lace
MCCM-LH9307	Thin lace
MCCM-LH9308	Thin lace
MCCM-LH9311	Thin lace
MCCM-LH9312	Thin lace
MCCM-LH9315	Thin lace
MCCM-LH9340	Thin lace
MCCM-LH9344	Thin lace
MCCM-LH9364	Thin lace
MCCM-LH9365	Thin lace
MCCM-LH9396	Thin lace
MCCM-LH9397	Thin lace
MCCM-LH9405	Thin lace
MCCM-LH9428	Thin lace
MCCM-LH9452	Thin lace
MCCM-LH9456	Thin lace
MCCM-LH9521	Thin lace
MCCM-LH9521 a/b	Thin lace
MCCM-LH9571	Thin lace
MCCM-LH14032	Thin lace
MCCM-LH14135	Thin lace
MCCM-LH14160	Thin lace

MCCM-LH14163	Thin lace
MCCM-LH14169 a/b	Thin lace
MCCM-LH14172 a/b	Thin lace
MCCM-LH14272	Thin lace
MCCM-LH15014	Thin lace
MCCM-LH15085	Thin lace
MCCM-LH15222	Thin lace
MCCM-LH15242	Thin lace
MCCM-LH15245	Thin lace
MCCM-LH15271	Thin lace
MCCM-LH15344	Thin lace
MCCM-LH15366	Thin lace
MCCM-LH15413 a/b	Thin lace
MCCM-LH15495	Thin lace
MCCM-LH15506	Thin lace
MCCM-LH15525 a/b	Thin lace
MCCM-LH15614	Thin lace
MCCM-LH15729	Thin lace
MCCM-LH15757	Thin lace
MCCM-LH15767	Thin lace
MCCM-LH15817	Thin lace
MCCM-LH15881	Thin lace
MCCM-LH15919	Thin lace
MCCM-LH15941	Thin lace
MCCM-LH15968	Thin lace
MCCM-LH16541	Thin lace
MCCM-LH16548 a/b	Thin lace
MCCM-LH16557 a/b	Thin lace
MCCM-LH16564 2plates	Thin lace
MCCM-LH16585 a/b	Thin lace
MCCM-LH16590 a	Thin lace
MCCM-LH16606 a/b	Thin lace
MCCM-LH16759 a/b	Thin lace
MCCM-LH20155	Thin lace
MCCM-LH20157 a/b	Thin lace
MCCM-LH20187 a/b	Thin lace
MCCM-LH20197 a/b	Thin lace
MCCM-LH20209	Thin lace
MCCM-LH20253	Thin lace
MCCM-LH20304	Thin lace
MCCM-LH20348 a/b	Thin lace
MCCM-LH20572 a/b	Thin lace
MCCM-LH21034	Thin lace
MCCM-LH21037	Thin lace
MCCM-LH21039	Thin lace
MCCM-LH21074	Thin lace
MCCM-LH21094	Thin lace
MCCM-LH21095	Thin lace
MCCM-LH21109 a/b	Thin lace
MCCM-LH21131 a/b	Thin lace

MCCM-LH21149	Thin lace
MCCM-LH21166	Thin lace
MCCM-LH21172	Thin lace
MCCM-LH21204 a/b	Thin lace
MCCM-LH21274	Thin lace
MCCM-LH21292	Thin lace
MCCM-LH21309 a/b	Thin lace
MCCM-LH21357 a/b	Thin lace
MCCM-LH21371	Thin lace
MCCM-LH21377 a/b	Thin lace
MCCM-LH21382 a/b	Thin lace
MCCM-LH21394 a/b	Thin lace
MCCM-LH21403	Thin lace
MCCM-LH21405	Thin lace
MCCM-LH21408	Thin lace
MCCM-LH21421 a/b	Thin lace
MCCM-LH21426 a/b	Thin lace
MCCM-LH21438	Thin lace
MCCM-LH21448	Thin lace
MCCM-LH28194 a/b	Thin lace
MCCM-LH32343	Thin lace
MCCM-LH35426	Thin lace
MCCM-LH35428 a/b	Thin lace
MCCM-LH36094	Thin lace
MCCM-LH-GQ15-003	Thin lace
MCCM-LH-GQ15-007	Thin lace
MCCM-LH-LI15-007	Thin lace
MCCM-LH-LI15-014 (1)	Thin lace
MCCM-LH-LI15-016	Thin lace
MCCM-LH-LI15-017	Thin lace
MCCM-LH-LI15-018	Thin lace
MCCM-LH-LI15-025	Thin lace
MCCM-LH-LI15-028	Thin lace
MCCM-LH-LI15-034	Thin lace
