# Supplementary material for: Exceptional coprolite association from the Early Cretaceous continental Lagerstätte of Las Hoyas, Cuenca, Spain
Source: PLoS One. 2018 May 23;13(5):e0196982. doi: 10.1371/journal.pone.0196982 (PMC5965836; doi:10.1371/journal.pone.0196982)
Supplement: S2 Table — (PDF) [file pone.0196982.s002.pdf]

**Spiral** (Fig 6 A-B) With spiral marks, in reference to oblique strips on the surface of the coprolite, which vary in number. The width of the strips is regular; strips may occupy the entire coprolite.	**Circular** (Fig 6 C-D) They are rather flat, lentil-like, without relevant volume (no spheroidal or disc-shaped). They are mostly 'imperfect' circles with roughly rounded outline.	**Irregular** (Fig 6 E-G) This assembly includes all coprolites that have not been grouped as a defined morphotype, including those presenting circumvolutions, an unclear major dimension, and/or a mosaic of shapes.	**Elongated** (Fig 6 H-I) They show a straight longitudinal axis with flat ends, the ensemble conferring a roughly rectangular shape. They do not have noticeable volume and are usually flat.	**Rosary** (Fig 7 A) They present constrictions in the coprolite matrix throughout the longitudinal axis. These constrictions separate a series of wide bumps joined by narrow tracts. The number of constrictions is consistently greater than two. It can be suggested that these bumps and constrictions could indicate sphincter contractions during defecation.	**Ellipsoidal** (Fig 7 B-D) These coprolites show no special elongation, but distinct minor and major axes: proportion between axes is usually 1/2 to 1/3. The diameter is bigger at mid-length. These coprolites are not as flat as those of the circular morphotype.
**Overall shape:** Elongated. **Outline:** Straight. **Diameter:** 2–6 mm. **Length:** 9–12 mm. **End shapes:** Similar shape within individual coprolites but varies among different spiral coprolites. **Coprolite matrix colour:** Medium or dark. **Density of inclusions** Category 1. **Kind of inclusions:** Some specimens with no visible inclusions, other specimens with small inclusions impossible to identify. **Analogous shapes**: Spiral-F2	**Overall shape:** Rounded. **Outline:** Curved and irregular. **Diameter:** 7.5–100 mm. **Length:** Not applicable. **End shapes:** No defined ends. **Coprolite matrix colour:** Light. **Density of inclusions:** Categories 1 to 2. **Kind of inclusions:** Some remains of bones (fish vertebrae and scales). **Analogous shapes:** Subrounded- none	**Overall shape:** Undefined. **Outline:** A variety of possible combinations. **Diameter:** (if applicable) 2–39 mm. **Length:** 6–82 mm. **End shapes:** Not applicable. **Coprolite matrix colour:** Light or dark. **Density of inclusions:** Categories 1 to 3. **Kind of inclusions:** Different on each particular specimen: no remains, some plant remains, arthropod remains, and/or fish scales. **Analogous shapes:** Irregular -none	**Overall shape:** Rectangular. **Outline:** Straight. **Diameter:** 2–23 mm. **Length:** 6–29 mm. **End shapes:** Isopolar: flat ends. **Coprolite matrix colour:** Light to medium **Density of inclusions:** Categories 1 to 4. **Kind of inclusions:** Fish scales and thin bony remains. **Analogous shapes:** None-none	**Overall shape:** Segmented into bumps. **Outline:** Sinuous. **Diameter:** 1–5 mm. **Length:** 12.5–24 mm. **End shapes:** Isopolar: rounded ends. **Coprolite matrix colour:** Medium. **Density of inclusions:** Categories 1 to 3. **Kind of inclusions:** Thin bony remains and thick elements that could be scales or other bones. **Analogous shapes:** None-none	**Overall shape:** Roughly ovoid. **Outline:** Straight **Diameter:** 1.5–32 mm. **Length:** 4–75 mm. **End shapes:** Isopolar: rounded ends. **Coprolite matrix colour:** Light, medium or dark. **Density of inclusions:** Categories 1 to 4 **Kind of inclusions:** Depends on the specimen studied: No inclusions, bones, fish scales or vegetal remains. **Analogous shapes:** Cylindrical short-B1.

**Cylinder** (Fig 7 E-G)<br>The width throughout the longitudinal axis of the coprolite is roughly constant. Some specimens may have a comparatively wider diameter and consequently more volume, showing a stout and dense condition.	**Bump-headed lace** (Fig 8 A-B)<br>In reference to one of the ends, which shows a big bulge in comparison with the other end. The bulge is at least twice as wide as the rest of the coprolite.	**Fir-tree** (Fig 8 C)<br>In reference to the sequence of 'bumps' that decrease progressively from a wide to a very narrow end. There are two to four constrictions separating those bumps.	**Cone** (Fig 8 D-E)<br>The main character is that the diameter increases throughout the longitudinal axis, without constrictions. Their length is at least twice their width. One of the ends coincides with the maximum diameter of the coprolite, the other end with the minimum diameter.	**Straight lace** (Fig 8 F-G)<br>Longitudinal axis long and straight, unfolded, with a roughly similar diameter throughout its length. Length can be 4-10 times the corresponding diameter.	**Thin lace** (Fig 8 H-I)<br>Folded onto themselves, as their length is 10 times their width. They have a roughly identical diameter throughout their length.
**Overall shape:** Elongated, with notable volume.<br>**Outline** Straight to slightly curved.<br>**Diameter:** 1.5–20 mm.<br>**Length:** 8–100 mm.<br>**End shapes:** Isopolar: rounded ends.<br>**Coprolite matrix colour:** Light, medium or dark.<br>**Density of inclusions:** Categories 1 to 3.<br>**Kind of inclusions:** Thread-like bony elements (maybe scales embedded perpendicularly in the coprolite matrix) and thick bones.<br>**Analogous shapes:** Cylindrical-A4.	**Overall shape:** Elongated cord with a distinct bulge.<br>**Outline:** Sinuous.<br>**Diameter:** 0.5–5 mm.<br>**Length:** 8–46 mm.<br>**End shapes:** Anisopolar: large bulge at one end.<br>**Coprolite matrix colour:** Light, medium or dark.<br>**Density of inclusions:** Categories 2 to 4.<br>**Kind of inclusions:** Thread-like bony elements (probably scales embedded perpendicularly in the coprolite matrix) and rings (probably tiny fish vertebrae embedded in the coprolite matrix).<br>**Analogous shapes**: None-none.	**Overall shape:** Triangular.<br>**Outline:** Sinuous (more or less regular).<br>**Diameter:** 1–15 mm.<br>**Length:** 12–27 mm.<br>**End shapes:** Anisopolar: the smaller end is rounded and the other is almost straight or slightly bent.<br>**Coprolite matrix colour:** Light or medium.<br>**Density of inclusions:** Categories 1 to 4.<br>**Kind of inclusions:** Thin bony remains, big bones and scales.<br>**Analogous shapes:** None-none	**Overall shape:** Cone to tear-drop.<br>**Outline:** Straight.<br>**Diameter:** 1.5–15 mm.<br>**Length:** 3–24 mm.<br>**End shapes:** Anisopolar: the smaller end can be sharp or a bit rounded and the other one is almost straight or slightly bent.<br>**Coprolite matrix colour:** Light, medium or dark.<br>**Density of inclusions:** Categories 1 to 3.<br>**Kind of inclusions:** Bones, fish scales, some possible ostracods.<br>**Analogous shapes:** Cylindrical-B2.	**Overall shape:** Elongated cord<br>**Outline:** Sinuous.<br>**Diameter:** 1.5–4 mm.<br>**Length:** 12–55 mm.<br>**End shapes:** Anisopolar: one end always rounded and the other end flat to sharp.<br>**Coprolite matrix colour:** Light, medium or dark.<br>**Density of inclusions:** Categories 2 to 4<br>**Kind of inclusions:** The most abundant remains are thick bones, scales and thin bony fragments.<br>**Analogous shapes:** Thin Linear.	**Overall shape:** Ribbon-like.<br>**Outline:** Sinuous.<br>**Diameter:** 1–4 mm (up to 14 mm).<br>**Length:** 12–90 mm (up to 150 mm).<br>**End shapes:** Anisopolar. One end sharp the other flat or rounded.<br>**Coprolite matrix colour:** Light, medium or dark.<br>**Density of inclusions:** Categories 2 to 4<br>**Kind of inclusions:** Thread-like structures (probably scales embedded in coprolite matrix), rings (probably tiny fish vertebrae embedded in coprolite matrix) and thin bony remains (some of them seem to be segmented fin rays).<br>**Analogous shapes:** None-none.
